# The efficacy of resveratrol in the treatment of liver fibrosis: a systematic review and meta-analysis of preclinical studies

**DOI:** 10.3389/fnut.2025.1606603

**Published:** 2025-09-19

**Authors:** Dehua Luo, Zhoubiao Shang, Qingying He, Jianlong Ke, Qiqi Xian, Shunxin Dai, Sheng Sun, Shaoquan Xiong

**Affiliations:** ^1^Department of Medical Oncology, Hospital of Chengdu University of Traditional Chinese Medicine, Chengdu, China; ^2^School of Clinical Medicine, Chengdu University of Traditional Chinese Medicine, Chengdu, China; ^3^School of Acupuncture and Tuina, Chengdu University of Traditional Chinese Medicine, Chengdu, China

**Keywords:** resveratrol, liver fibrosis, preclinical studies, meta–analysis, systematic review

## Abstract

**Objective:**

To evaluate the effects and underlying mechanisms of resveratrol—a plant-derived polyphenol abundantly found in natural dietary sources such as grapes and blueberries—on the amelioration of liver fibrosis.

**Methods:**

Data were obtained from a systematic review of 46 animal studies identified across seven databases. Study quality was assessed using the SYRCLE tool for risk of bias. Meta-analysis was performed with Stata 17.0. Outcome measures included collagen deposition, hydroxyproline content, extracellular matrix components (HA, LN, CIV, PIIINP), key fibrogenic mediators (TGF-β, α-SMA, Col1α1), liver function markers (albumin, ALT, AST, ALP), as well as inflammatory and oxidative stress indicators.

**Results:**

Resveratrol markedly attenuated collagen deposition and reduced hydroxyproline levels, a central marker of fibrotic progression. It significantly inhibited the accumulation of extracellular matrix components and modulated profibrotic mediators. Improvement in liver function was indicated by elevated albumin levels and decreased activities of ALT, AST, and ALP. Mechanistically, resveratrol exerted dual modulation through the following pathways: Inflammatory pathways: downregulation of IL-6 and TNF-α; Oxidative stress responses: enhancement of SOD and GSH activities, accompanied by reduction in MDA levels.

**Conclusion:**

Resveratrol significantly alleviates liver fibrosis in animal models via anti-inflammatory and antioxidant mechanisms. However, translation to clinical practice requires further validation owing to interspecies differences and notable heterogeneity across included studies. Standardized preclinical study designs and cross-species mechanistic investigations are warranted to support future clinical applications.

**Systematic review registration:**

The registered website: https://www.crd.york.ac.uk/PROSPERO/view/CRD42025633941.

## 1 Introduction

Liver fibrosis (LF) represents a pathological state characterized by excessive extracellular matrix deposition, primarily collagen, secondary to chronic hepatic injury. This process disrupts hepatic architecture, progressively impairing function and potentially advancing to cirrhosis, liver failure, or hepatocellular carcinoma (1). Affecting 2–19% of the global population (2, 3), chronic liver diseases impact approximately 1.5 billion individuals (4). LF exacerbates complications including ascites, portal hypertension, hepatic encephalopathy, liver failure, and elevates the risk of carcinogenesis, imposes substantial burdens on healthcare systems and societies (5, 6). Primary etiologies encompass chronic viral hepatitis (hepatitis B/C), alcohol-related liver damage, non-alcoholic fatty liver disease (NAFLD), and autoimmune hepatic disorders (7, 8).

The pathogenesis of LF involves a complex interplay among inflammatory responses, activation of hepatic stellate cells (HSC), and dysregulated extracellular matrix (ECM) turnover, driven primarily by key signaling pathways including transforming growth factor-*β* (TGF-β)/Smad and Wnt/β-catenin (9, 10). Despite advances in understanding LF’s molecular mechanisms, current therapeutic options remain limited. Antifibrotic drugs, such as pirfenidone and nintedanib, demonstrate limited antifibrotic efficacy, significant side effects, and variable effectiveness in heterogeneous disease presentations (11, 12). Liver transplantation, provides a curative approach, is constrained by donor shortages, high costs, and post-transplant complications (13). Therefore, there is an urgent need to identify novel therapeutic agents with improved safety and efficacy profiles that can prevent or reverse LF.

**Figure 1:**
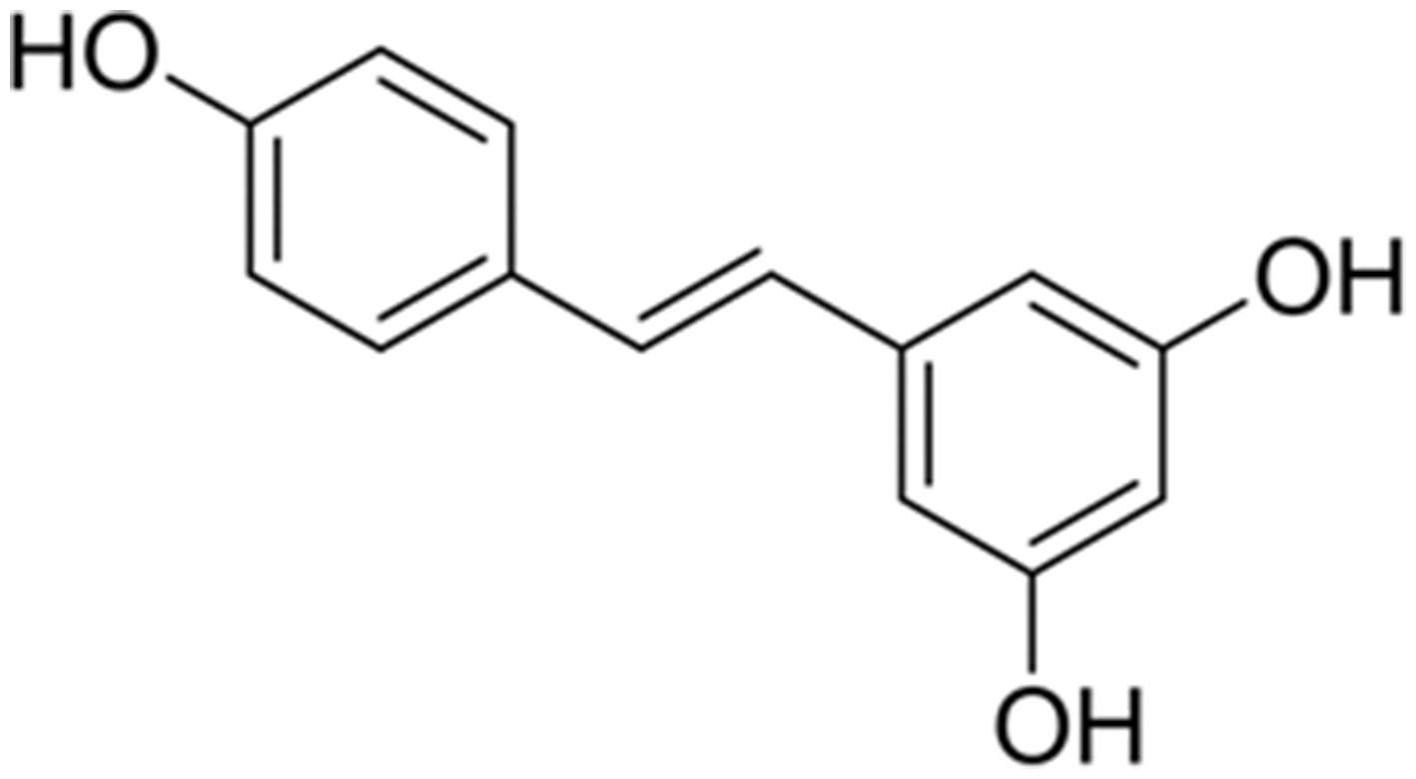
The chemical structure of resveratrol.

Resveratrol (3,5,4′-trihydroxystilbene, Figure 1), a natural polyphenolic compound derived from botanical sources including grapes, berries, and peanuts with particularly high concentrations in red wine (14). It has been consumed as part of the human diet for centuries and is generally recognized as safe (GRAS) by regulatory agencies, making it an attractive candidate for therapeutic applications (15). Beyond nutritional functions, resveratrol exhibits therapeutic promise for cardiovascular, metabolic, and oncological disorders via its antioxidant, anti-inflammatory, and anti-fibrotic actions (16, 17). In recent years, preclinical studies have highlighted the potential of resveratrol in attenuating LF in animal models. For instance, resveratrol has been shown to HSC activation, reduce oxidative stress, and modulate key fibrogenic pathways such as TGF-*β*/Smad and nuclear factor kappa-B (NF-Κb) (18, 19). Despite these promising findings, clinical translation faces significant bottlenecks. First, the bioavailability of resveratrol is extremely low (<1% following oral administration). Although nano-delivery systems (e.g., liposomes) or structural modifications (e.g., resveratrol derivatives) can enhance stability, the long-term toxicity and industrial-scale production feasibility still require verification (20, 21). Second, substantial heterogeneity exists in preclinical studies. Differences in modeling methods, dosage regimens, and efficacy evaluation criteria among various animal models compromise the comparability of results. Integrating data through meta-analysis is urgently needed to clarify the dose–response relationship. Finally, the discrepancy between animal models and human pathology limits predictive value. Existing models are primarily based on single causes, whereas human LF is often driven by the interaction of multiple factors. Interspecies differences may also overestimate resveratrol’s *in vivo* effects. Although anatomical and physiological differences exist between animal models and humans, animal research remains crucial for exploring the pathophysiology of human diseases.

## 2 Methods

This study was conducted in accordance with the Preferred Reporting Items for Systematic Reviews and Meta-Analyses (PRISMA) guidelines (22). The protocol was registered in the PROSPERO International Prospective Register of Systematic Reviews (registration number: CRD42025633941).

### 2.1 Search strategy

A comprehensive literature search was performed to identify all relevant preclinical animal studies investigating the efficacy of resveratrol in preventing LF. The following electronic databases were searched: Web of Science, Embase, PubMed, China Biology Medicine (CBM), China National Knowledge Infrastructure (CNKI), Wanfang Database (WF), and China Science Journal Database (VIP). The search was limited to studies published before December 31, 2024, to ensure the inclusion of the most recent evidence. Additionally, manual searches of reference lists from included studies and relevant reviews were conducted to identify potentially eligible studies that might have been missed in the electronic database searches. The search strategy employed a combination of Medical Subject Headings (MeSH) terms and free-text words to maximize the sensitivity and specificity of the search (Supplementary Table 1).

### 2.2 Eligibility criteria

The inclusion criteria for this study were defined based on the PICO framework to ensure the selection of relevant and high-quality preclinical animal studies. Animal: Studies utilizing animal models of LF were included, regardless of the species, sex, age, or weight of the animals. Both induced (e.g., chemically induced, diet-induced) and genetic models of LF were considered eligible. Intervention: Studies in which animals were treated with resveratrol, regardless of the dose, duration, frequency, or route of administration, were included. Comparison: Studies must have included a control group receiving either an equivalent vehicle, physiological saline, or no treatment. Outcomes: Studies reporting outcomes related to the protective effects of resveratrol on LF were included. Primary outcomes of interest included histopathological changes in LF (e.g., collagen deposition, degree of tissue fibrosis), LF progression markers [e.g., hydroxyproline (HYP), *α*-smooth muscle actin (α-SMA)], TGF-*β*, and inflammatory cytokine levels [e.g., interleukin-6 (IL-6), tumor necrosis factor-α (TNF-α)]. Secondary outcomes such as liver function biomarkers (e.g., ALT, AST) and oxidative stress markers [e.g., malondialdehyde (MDA), superoxide dismutase (SOD), and glutathione (GSH)] were also considered.

Exclude the following studies: (1) Study Type: Clinical studies, *in vitro* experiments, and computer simulation studies were excluded. (2) Intervention: Studies without a control group or those in which the treatment group received a combination of resveratrol and other therapeutic interventions were excluded. (3) Duplicate Publications: In cases of duplicate publications, the most recent or comprehensive study was retained, and earlier versions were excluded.

### 2.3 Data extraction

Data extraction was conducted independently by two researchers (QH and JK) to ensure accuracy and minimize bias. The process involved the following steps: (1) Initial Screening: Titles and abstracts of all retrieved studies were screened to exclude irrelevant publications. Studies that did not meet the inclusion criteria were removed at this stage. (2) Full-Text Review: The remaining studies were subjected to a full-text review to assess their eligibility based on the predefined inclusion and exclusion criteria. (3) Data Collection: For studies meeting the inclusion criteria, the following information was extracted: Publication Details: Authors and year of publication. Animal Characteristics: Species, sex, age, weight, and sample size. LF Model: Method used to induce LF (e.g., chemical induction, diet-induced, genetic models). Intervention Details: Resveratrol administration parameters, including dose, duration, frequency, route of administration, and control group treatment. Outcome Measures: Data on histopathological changes, LF progression markers, inflammatory cytokine levels, liver function biomarkers, and oxidative stress markers. If outcome data were presented only in graphical form, attempts were made to contact the corresponding authors to obtain raw data. If raw data were unavailable, graphical data were digitized using WebPlotDigitizer 4.5,^1^ a validated tool for extracting numerical data from graphs. For studies reporting multiple data points due to varying doses or time points, data from the group receiving the maximum effective dose or the latest effective time point were extracted for meta-analysis. Any disagreements between the two researchers during data extraction were resolved through discussion and, if necessary, consultation with a third researcher (SX) to reach a consensus.

### 2.4 Quality assessment

The methodological quality and risk of bias of the included studies were independently assessed by two reviewers (ZS and QX) using the Systematic Review Center for Laboratory Animal Experimentation (SYRCLE) risk of bias tool (23). This tool is specifically designed to evaluate the risk of bias in animal studies and includes the following domains: sequence generation, baseline characteristics, allocation concealment, random housing, blinding of experimentalists, random outcome assessment, blinding of outcome assessors, incomplete outcome data, selective outcome reporting, other sources of bias. Each domain was assessed and categorized as “yes” (low risk of bias), “no” (high risk of bias), or “unclear” (uncertain risk of bias) based on the information provided in the studies. Discrepancies between the two reviewers were resolved through discussion, and if consensus could not be reached, a third reviewer (SX) was consulted to make the final decision.

### 2.5 Statistical analysis

Statistical analyses were performed using STATA software (version 17.0). For continuous outcome measures, the overall effect size was expressed as the standardized mean difference (SMD) with 95% confidence intervals (CIs). A *p*-value of < 0.05 was considered statistically significant. Heterogeneity among studies was assessed using the I^2^ statistic, which quantifies the proportion of total variability in effect estimates attributable to heterogeneity rather than chance. The following thresholds were used to interpret the I^2^ values: I^2^ ≤ 50%: Low to moderate heterogeneity, indicating that a fixed-effects model was appropriate for meta-analysis. I^2^ > 50%: Substantial heterogeneity, prompting further investigation through sensitivity analysis and subgroup analysis to identify potential sources of heterogeneity. If significant heterogeneity persisted and could not be resolved, a random-effects model was applied to account for between-study variability. Sensitivity analysis was conducted by sequentially excluding individual studies to evaluate their impact on the overall effect size and heterogeneity. Subgroup analyses were performed based on predefined factors, such as animal species, LF induction method, resveratrol dosage, and treatment duration, to explore potential sources of heterogeneity. Publication bias was assessed using Egger’s linear regression test and Begg’s rank correlation test. If evidence of publication bias was detected (*p* < 0.05), the trim-and-fill method was employed to adjust for potential bias and estimate the corrected effect size.

## 3 Results

### 3.1 Study selection

**Figure 2:**
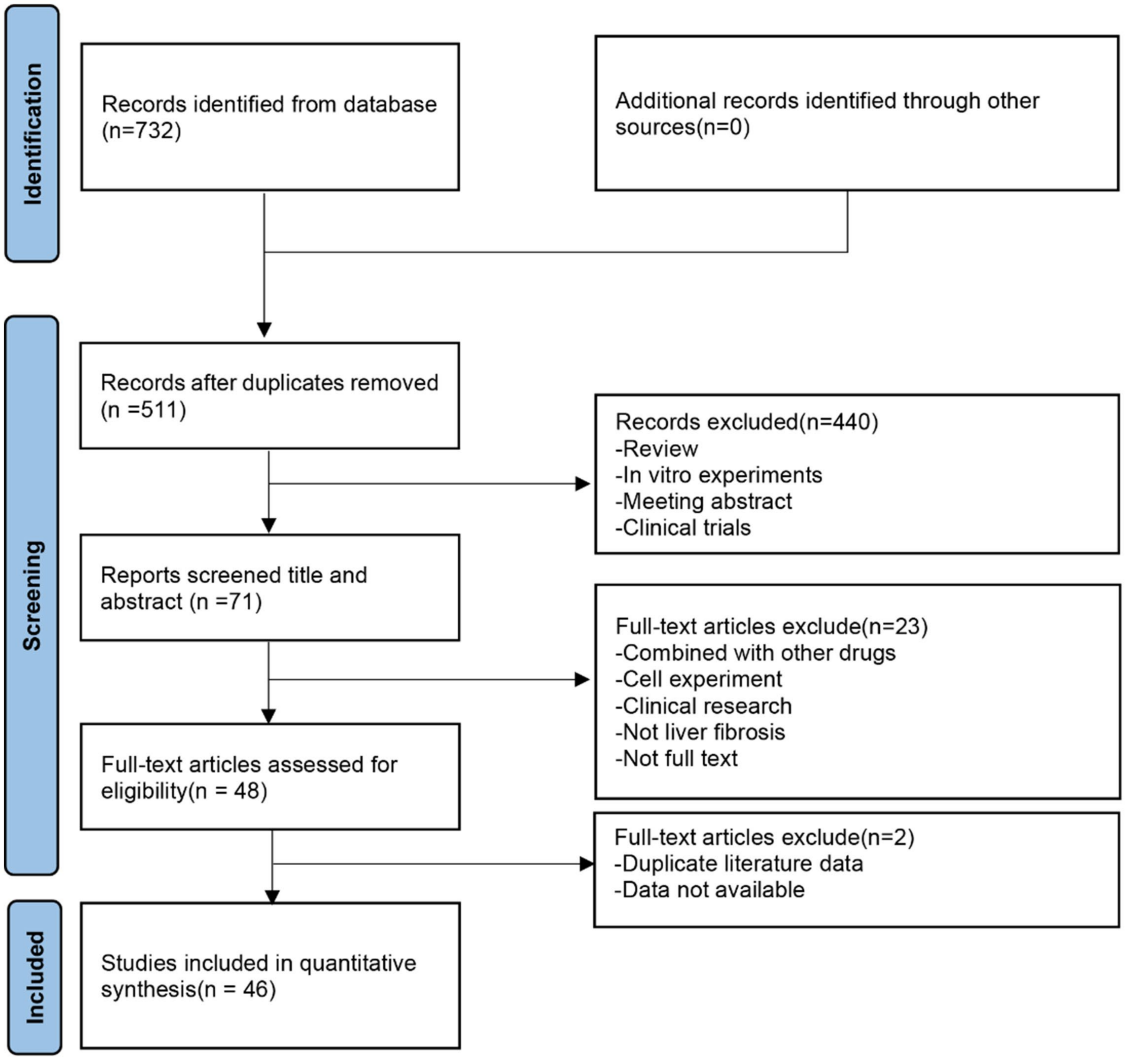
Flow diagram of the study selection process.

A total of 732 potentially relevant articles were retrieved from seven online databases, including PubMed (24), Embase (295), Web of Science (233), CNKI (25), CBM (26), Wanfang (27), and VIP (19). After removing duplicates, 511 articles remained. Subsequently, 440 articles were excluded based on the screening of titles and abstracts. Following a full-text review, an additional 25 articles were excluded, resulting in the final inclusion of 46 studies. The flow diagram of the study selection process is presented in Figure 2.

### 3.2 Characteristics of included studies

The 46 included studies involved a total of 751 animals, with 375 in the treatment groups and 376 in the control groups. Among these, 11 studies used 172 Sprague–Dawley (SD) rats (26, 28–37), 8 studies used 124 C57BL/6 J mice (25, 38–44), 11 studies used 229 Wistar rats (45–55), 5 studies used 46 Balb/c mice (27, 56–59), 3 study used 40 Kunming mice (60–62), 2 studies used 32 Swiss mice (24, 63), 1 study used CD-1 mice (64), and 1 study used 20 Japanese quails (65). Additionally, 4 studies involved 86 rats of unspecified strains (66–69). Regarding the sex of the animals, 39 studies used male animals, 1 study used female animals, 4 studies used both male and female animals, and 2 studies did not specify the sex. The age of the animals was reported in 26 studies, and the weight was described in 36 studies. In terms of resveratrol administration, 34 studies utilized oral gavage or intragastric administration, 11 studies employed intraperitoneal injection, and 1 study used tail vein injection. For LF assessment, 24 studies reported histopathological changes in LF, 18 studies measured TGF-*β* levels, 21 studies evaluated *α*-SMA expression, 19 studies assessed type I collagen, 18 studies measured HYP, 6 studies reported hyaluronic acid (HA), 5 studies evaluated type III procollagen N-terminal propeptide (PIINP), 3 studies measured collagen type IV (COL-IV), and 6 studies reported laminin (LN). Regarding liver function, 37 studies reported ALT levels, 31 studies reported AST levels, 11 studies measured albumin (ALB), and 12 studies evaluated alkaline phosphatase (ALP). For oxidative stress markers, 21 studies reported MDA levels, 10 studies measured GSH, and 15 studies evaluated SOD. In terms of inflammatory cytokine levels, 13 studies reported TNF-*α*, 6 studies measured IL-6, 5 studies assessed IL-1β, and 6 studies evaluated NF-κB. Detailed characteristics of the included studies are presented in Table 1.

**Table 1:** Basic characteristics of the included studies.

Study (year)	Species (sex, age, *n* = treatment/model group, weight)	Modeling method	Resveratrol intervention (administration drug dose, duration)	Outcomes
Li et al. (60)	Kunming mice (/, /, 7/7, 22 ± 2 g)	Liver Fibrosis Induced by Long-Term Inorganic Mercury Exposure	The mice were given with aqueous solution containing 100 mg/L HgCl2 and gavage with 100 mg/kg body weight resveratrol once a day for 6 weeks	1. HYP 2. ALT 3. AST 4. TGF-β 5. α-SMA 6. Col1α1 7. MDA 8. GSH 9. SOD 10. TNF-α
Mostafa et al. (63)	Swiss albino mice (male, /, 12/12, 20–25 g)	*Schistosoma mansoni*-induced liver fibrosis	The mice were orally administered 20 or 100 mg/kg of resveratrol daily for 4 weeks	1. ALT 2. AST 3. TGF-β 4. α-SMA 5. Col1α1 6. ALB 7. ALP 8. TNF-α
Kabir et al. (25)	C57BL/6 J mice (male, 10 weeks, 8/8, 23 g)	High-fat choline-deficient diet-induced hepatic fibrosis	The mice were orally administered 150 mg/kg of resveratrol daily for 12 weeks	1. Collagen deposition 2. ALT 3. AST 4. α-SMA 5. Col1α1
Li et al. (56)	Balb/c mice (male, 6–7 weeks, 5/5, /)	Liver fibrosis induced by CCl4	The mice were treated with 30 mg/kg resveratrol daily by gavage, followed by intraperitoneal injection of 0.5 μL/g CCl4 twice a week for 4 weeks	1. Collagen deposition 2. HYP 3. ALT 4. AST 5. α-SMA 6. Col1α1 7. ALP
Ma et al. (28)	Sprague–Dawley rats (male, 3–4 weeks, 6/6, 200-250 g)	Liver fibrosis induced by CCl4	The rats were treated with 10, 20, 30 mg/kg resveratrol daily by gavage for 12 weeks	1. Collagen deposition 2. ALT 3. AST 4. TGF-β 5. ALP 6. TNF-α 7. IL-6
Wang et al. (61)	Kunming mice (male, 6–7 weeks, 10/10, 20 ± 2 g)	Iron overload-induced liver fibrosis	The mice were treated with 25, 50, 100 mg/kg resveratrol daily by gavage for 12 weeks	1. Collagen deposition 2. HYP 3. ALT 4. AST 5. α-SMA 6. Col1α1 7. MDA 8. GSH 9. SOD 10. TNF-α 11. IL-6
Ebrahim et al. (66)	Albino rats (male, /, 6/6, 180–200 g)	Thioacetamide-induced liver fibrosis	Rats were treated with resveratrol suspension (20 mg/kg, orally) daily for 10 weeks	1. Collagen deposition 2. ALT 3. AST 4. α-SMA 5. TNF-α
Lianget al (38).	C57BL/6 J mice (male, 8 weeks, 5/5, /)	Liver fibrosis induced by CCl4	The mice in the Res group were injected intraperitoneally with 10% CCl4 and Res (100 mg/kg/d) daily for 4 weeks	1. HYP 2. ALT 3. AST 4. Col1α1 5. ALB
Hung et al. (64)	CD-1 mice (male, 5 weeks, 10/10, /)	Liver fibrosis induced by CCl4	The mice were treated with 30 mg/kg resveratrol daily by gavage for 8 weeks	1. Collagen deposition 2. ALT 3. AST 4. TGF-β 5. α-SMA 6. Col1α1 7. ALP
Li et al. (65)	Japanese quails (male, 21 days, 10/10, 80 ± 15 g)	Liver fibrosis induced by deltamethrin	The quails were treated with 500 mg/kg resveratrol daily by gavage for 12 weeks	1. Collagen deposition 2. HYP 3. ALT 4. AST 5. TGF-β 6. α-SMA 7. Col1α1 8. MDA 9. GSH 10. SOD 11. TNF-α 12. LN
Shams Eldeen (45)	Wistar rats (male, /, 6/6, 150–200 g)	Cholestasis-induced liver fibrosis	The rats were orally administered 10,20,30 mg/kg of resveratrol daily for 3 weeks	1. Collagen deposition 2. ALT 3. AST 4. TGF-β 5. MDA 6. GSH
Zhu et al. (29)	Sprague–Dawley rats (male, 6–8 weeks, 6/6, 250 ± 30 g)	Liver fibrosis induced by CCl4	The rats were treated with 40, 120, 200 mg/kg resveratrol daily by gavage for 4 weeks	1. Collagen deposition 2. HYP 3. ALT 4. AST 5. TGF-β 6. α-SMA 7. ALB 8. MDA 9. SOD 10. HA 11. PcIII 12. LN
Chen et al. (57)	Balb/c mice (male, 6–8 weeks, 5/5, /)	Schistosoma-induced liver fibrosis	Mice were treated with a resveratrol suspension (400 mg/kg/d) for 3 days by gastric gavage at 6 weeks after infection	1. Collagen deposition 2. α-SMA 3. Col1α1
Mohseni et al. (46)	Wistar rats (male, 6 weeks, 5/5, 200 ± 15 g)	Liver fibrosis induced by CCl4	Mice were treated with a resveratrol suspension (10 mg/kg/d) for 8 weeks by gastric gavage	1. HYP 2. ALT 3. AST 4. Col1α1 5. ALP 6. MDA
Yu et al. (58)	Balb/c mice (male, 6 weeks, 6/6, 18–20 g)	Liver fibrosis induced by CCl4	Mice were injected intraperitoneally with resveratrol (400 mg/kg/d)	1. Collagen deposition 2. HYP 3. ALT 4. AST 5. TNF-α
Hessin et al. (47)	Albino Wistar rats (male, adult, 18/18, 200–250 g)	Thioacetamide-induced liver fibrosis	The rats were orally administered 30 mg/kg of resveratrol daily for 4 weeks	1. HYP 2. ALT 3. AST 4. ALB 5. MDA 6. GSH
Tanriverdi et al. (48)	Albino Wistar rats (male, 5 weeks, 10/10, 200–250 g)	Liver fibrosis induced by CCl4	Rats were injected intraperitoneally with resveratrol (1 mg/kg/d) for 6 weeks	1. Collagen deposition 2. TGF-β 3. α-SMA 4. GSH
Zhang et al (39)	C57BL/6 J mice (male, 8 weeks, 5/5, /)	Liver fibrosis induced by CCl4	The mice were treated with 20, 50 mg/kg resveratrol daily by gavage for 8 weeks	1. ALT 2. AST 3. α-SMA 4. Col1α1 5. TNF-α
Ahmad et al. (49)	Albino Wistar rats (male, 6–8 weeks, 5/5, 160 ± 10 g)	N0-nitrosodimethylamine-induced liver fibrosis	Rats were given three consecutive intraperitoneal injections of resveratrol (10 mg/kg, three days a week) for 3 weeks	1. Collagen deposition 2. HYP 3. ALT 4. AST 5. α-SMA 6. ALP 7. MDA 8. SOD
Pascoli et al. (50)	Wistar rats (male, /, 10/8, 50-75 g)	Liver fibrosis induced by CCl4	Rats were treated with a resveratrol suspension (20 mg/kg/d) for 2 weeks by gastric gavage	1. TGF-β 2. α-SMA 3. Col1α1 4. SOD
El-Agamy et al. (24)	Swiss albino mice (male, /, 6/6, 18 ± 2 g)	*Schistosoma mansoni*-induced liver fibrosis	Mice began drug treatment daily on the 28th day after infection and continued for 2 weeks	1. HYP 2. ALT 3. AST 4. ALB
Chan et al. (40)	C57BL/6 J mice (/, /, 6/6, /)	Cholestasis-induced liver fibrosis	Rats were injected intraperitoneally with resveratrol (40 mg/kg/d) for 7 days	1. ALT 2. AST 3. TGF-β 4. Col1α1 5. TNF-α 6. IL-6
Hong et al. (30)	Sprague–Dawley rats (male, 6 weeks, 6/6, /)	Dimethylnitrosamine-induced liver fibrosis	The rats were orally administered 10 mg/kg of resveratrol daily for 7 days	1. HYP 2. ALT 3. AST 4. TGF-β 5. α-SMA 6. Col1α1 7. ALP 8. MDA 9. SOD 10. TNF-α
Lee et al. (31)	Sprague–Dawley rats (male, /, 6/6, 140-160 g)	Dimethylnitrosamine-induced liver fibrosis	Rats were treated with a resveratrol suspension (20 mg/kg/d) for 4 weeks by gastric gavage	1. HYP 2. ALT 3. AST 4. ALB 5. ALP 6. MDA
Chávez (51)	Wistar rats (male, /, 15/15, 90–100 g)	Liver fibrosis induced by CCl4	The rats were orally administered 10 mg/kg of resveratrol daily for 8 weeks	1. Collagen deposition 2. ALT 3. TGF-β 4. ALP 5. MDA 6. GSH
Ran et al. (32)	Sprague–Dawley rats (male and female, /, 8/8, 80-120 g)	Arsenic Exposure-Induced Liver Fibrosis	Rats received daily treatment with resveratrol (20 mg/kg) for 36 weeks, 6 days a week	1. Collagen deposition 2. α-SMA 3. Col1α1 4. IL-6 5. HA 6. PcIII 7. C-IV 8. LN
Aykac et al. (26)	Sprague–Dawley rats (male, 5–6 week s, 8/8, 150-200 g)	Liver fibrosis induced by CCl4	Rats were injected intraperitoneally with resveratrol (1 mg/kg/d) for 10 weeks	1. Collagen deposition 2. ALT 3. AST 4. α-SMA 5. ALB 6. ALP
Rashidi et al. (52)	Wistar rats (male, /, 8/8, 180–200 g)	Liver fibrosis induced by high-fat diet	The rats were orally administered 100 mg/kg of resveratrol daily for 6 weeks	1. ALT 2. AST 3. TGF-β 4. TNF-α 5. IL-6
Dawood et al. (67)	Albino rats (male, /, 8/8, 180–200 g)	Thioacetamide-induced liver fibrosis	The rats were orally administered 20 mg/kg of resveratrol daily for 10 weeks	1. Collagen deposition 2. ALT 3. α-SMA 4. MDA 5. SOD
Yang et al. (33)	Sprague–Dawley rats (male and female, 5–6 weeks, 3/3, 200–250 g)	Liver fibrosis induced by CCl4	The rats were orally administered 300 mg/kg of resveratrol daily for 8 weeks	1. Collagen deposition 2. ALT 3. AST 4. TGF-β 5. α-SMA 6. MDA 7. GSH 8. SOD 9. TNF-α
Abdu and Al-Bogami (68)	Albino rats (male, /, 7/7, 90–116 g)	Dimethylnitrosamine -induced liver fibrosis	The rats were treated with 20 mg/kg resveratrol daily by gavage for 3 weeks	1. HYP 2. ALT 3. AST 4. ALB 5. ALP 6. MDA 7. GSH 8. SOD
Mukherjee and Ahmad (69)	Albino rats (male, 6–8 weeks, 5/5, 150–160 g)	Nitrosodiethylamine-induced liver fibrosis	Rats were injected intraperitoneally with resveratrol (10 mg/kg/d) for 2 weeks	1. MDA 2. SOD
Kessoku et al. (41)	C57BL/6 J mice (male, 8 weeks, 5/5, /)	Liver fibrosis induced by high-fat diet	Mice were orally administered 20 mg/kg of resveratrol daily for 4 weeks	1. Collagen deposition 2. ALT 3. α-SMA 4. Col1α1 5. TNF-α 6. IL-6
Zhang et al. (39)	C57BL/6 J mice (male, 8 weeks, 5/5, /)	Schistosoma-induced liver fibrosis	The rats were treated with 20 mg/kg resveratrol daily by gavage for 6 weeks	1. Collagen deposition 2. TGF-β
Que (43)	C57BL/6 J mice (male, adult, 10/10, /)	Liver fibrosis induced by CCl4	Mice were injected intraperitoneally with resveratrol (30 mg/kg/d) for 4 weeks	1. Collagen deposition 2. ALT 3. AST 4. α-SMA 5. Col1α1 6. MDA
Zou (34)	Sprague–Dawley rats (male, /, 8/8, 200 ± 20 g)	Liver fibrosis induced by CCl4	Resveratrol is injected into the tail vein of rats three times a week for 4 weeks	1. HYP 2. ALT 3. AST 4. ALB 5. MDA 6. SOD 7. HA 8. PcIII 9. C-IV 10. LN
Yan et al. (62)	Balb/c mice (male, 6 -8 weeks, 6/4, 20–22 g)	Liver fibrosis induced by CCl4	Mice were injected intraperitoneally with resveratrol (400 mg/kg/d) for 5 weeks	1. Collagen deposition
Li (27)	Balb/c mice (male, 6 weeks, 3/3, 18–22 g)	Liver fibrosis induced by CCl4	Mice were injected intraperitoneally with resveratrol (400 mg/kg/d) for 5 weeks	1. Collagen deposition
Feng (44)	C57BL/6 J mice (male, 8 weeks, 8/8, /)	Liver fibrosis induced by CCl4	Mice were intraperitoneally injected with resveratrol (30 mg/kg) twice a week for 8 weeks	1. Collagen deposition 2. ALT 3. AST
Chen (62)	Kunming mice (male, 6–7 weeks, 10/10, 20 ± 2 g)	Schistosoma-induced liver fibrosis	Mice were treated with 20 mg/kg resveratrol daily by gavage for 6 weeks	1. Col1α1 2. MDA 3. SOD
Wan (35)	Sprague–Dawley rats (male, /, 16/22, 250 ± 50 g)	Liver fibrosis induced by CCl4	Rats were treated with 200 mg/kg resveratrol daily by gavage for 13 weeks	1. ALT 2. TGF-β 3. ALB 4. HA 5. PcIII
Liu (36)	Wistar rats (male, /, 8/8, 130–160 g)	Dimethylnitrosamine -induced liver fibrosis	Rats were treated with 200 mg/kg resveratrol daily by gavage for 4 weeks	1. ALT 2. MDA 3. GSH 4. SOD 5. HA 6. LN
Lin (54)	Wistar rats (female, /, 9/8, 80–100 g)	Porcine serum immune-induced liver fibrosis	Rats were treated with 30 mg/kg resveratrol daily by gavage for 8 weeks	1. HYP 2. ALT 3. AST 4. TGF-β 5. ALP 6. MDA 7. SOD 8. HA 9. PcIII 10. C-IV 11. LN
Niu (36)	Sprague–Dawley rats (male, /, 8/8, 250 ± 30 g)	Liver fibrosis induced by CCl4	Rats were treated with 200 mg/kg resveratrol daily by gavage for 8 weeks	1. HYP 2. ALT 3. α-SMA 4. ALB 5. MDA 6. SOD 7. HA 8. PcIII
Lv (55)	Wistar rats (male and female, /, 10/10, 120-160 g)	Liver fibrosis induced by CCl4	Rats were treated with 100 mg/kg resveratrol daily by gavage for 6 weeks	1. HYP 2. ALT 3. AST 4. Col1α1 5. ALB 6. MDA
Qi (37)	Sprague–Dawley rats (male, /, 8/8, 180-200 g)	Dimethylnitrosamine -induced liver fibrosis	Rats were treated with 100 mg/kg resveratrol daily by gavage for 7 weeks	1. Collagen deposition 2. ALT 3. AST 4. TGF-β 5. Col1α1

HYP, hydroxyproline; ALT, alanine aminotransferase; AST, aspartate aminotransferase; α-SMA, α-smooth muscle actin; TGF-β, transforming growth factor-β; Col1α1, collagen type I alpha 1; MDA, malondialdehyde; SOD, superoxide dismutase; GSH, glutathione; IL-6, interleukin-6; TNF-α, tumor necrosis factor-α; HA, hyaluronic acid; LN, laminin; CIV, type IV collagen; PIIINP, type III procollagen N-terminal peptide; ALP, alkaline phosphatase; ALB, albumin.

### 3.3 Study quality

**Figure 3:**
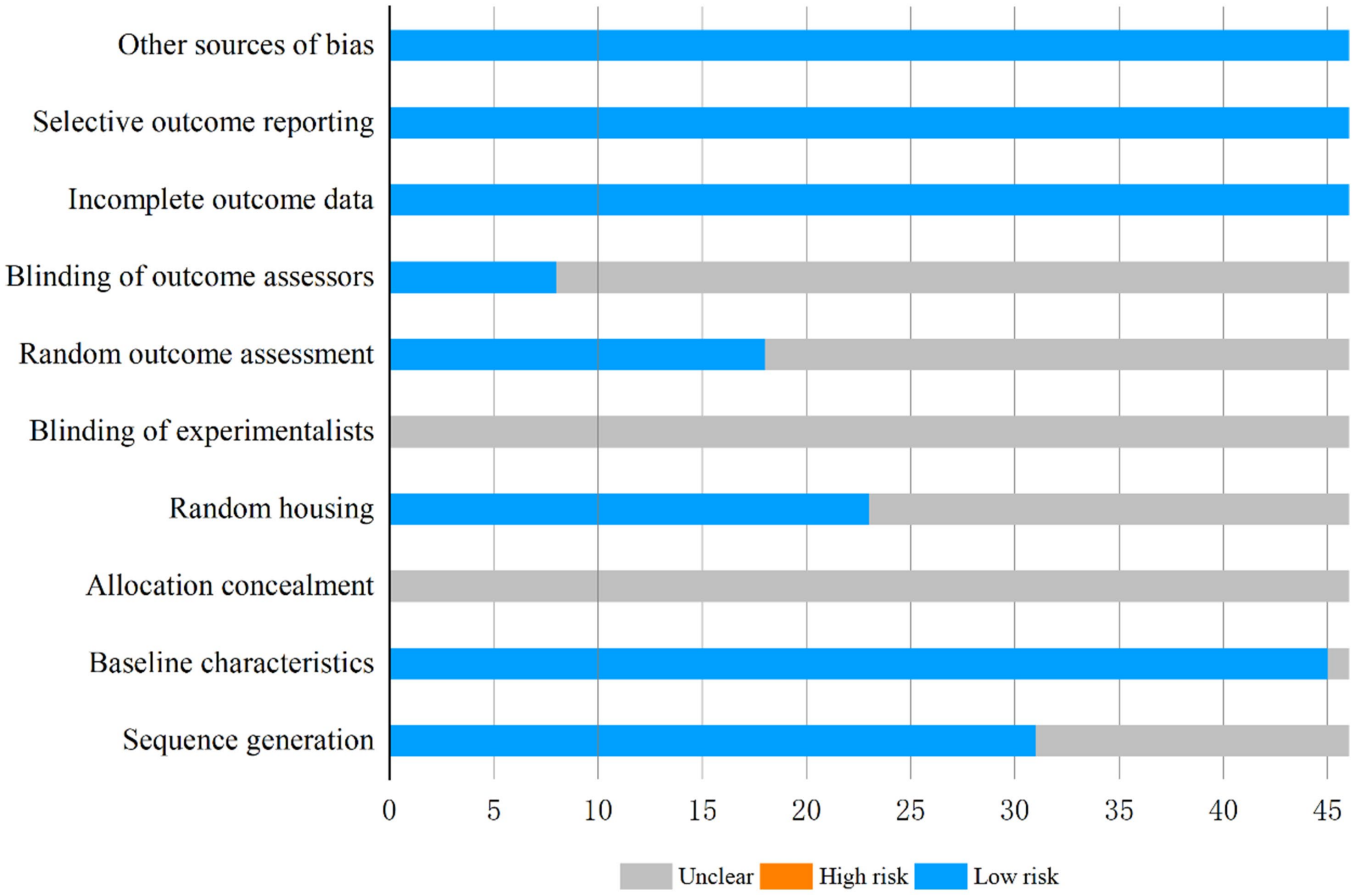
Risk of bias graph.

Quality assessment of the 46 included studies using the SYRCLE risk-of-bias tool revealed the following score distribution: 4 studies scored 4, 7, and 8 points each, with 14 and 17 studies attaining 5 and 6 points, respectively. Methodological quality analysis (Figure 3) demonstrated that baseline characteristics of experimental subjects were documented in 45 studies, among which 31 explicitly implemented animal randomization, 23 reported randomized housing, and 18 utilized randomized outcome assessment. Notably, while 8 studies declared blinded outcome evaluation, none provided specific details regarding allocation concealment or operator blinding. All included studies met low-risk criteria in three critical domains: data completeness, avoidance of selective reporting, and control of additional biases. Comprehensive evaluation data are compiled in Supplementary Table 2.

### 3.4 Effectiveness

#### 3.4.1 Primary outcomes

##### 3.4.1.1 The condition of liver fibrosis

**Figure 4:**
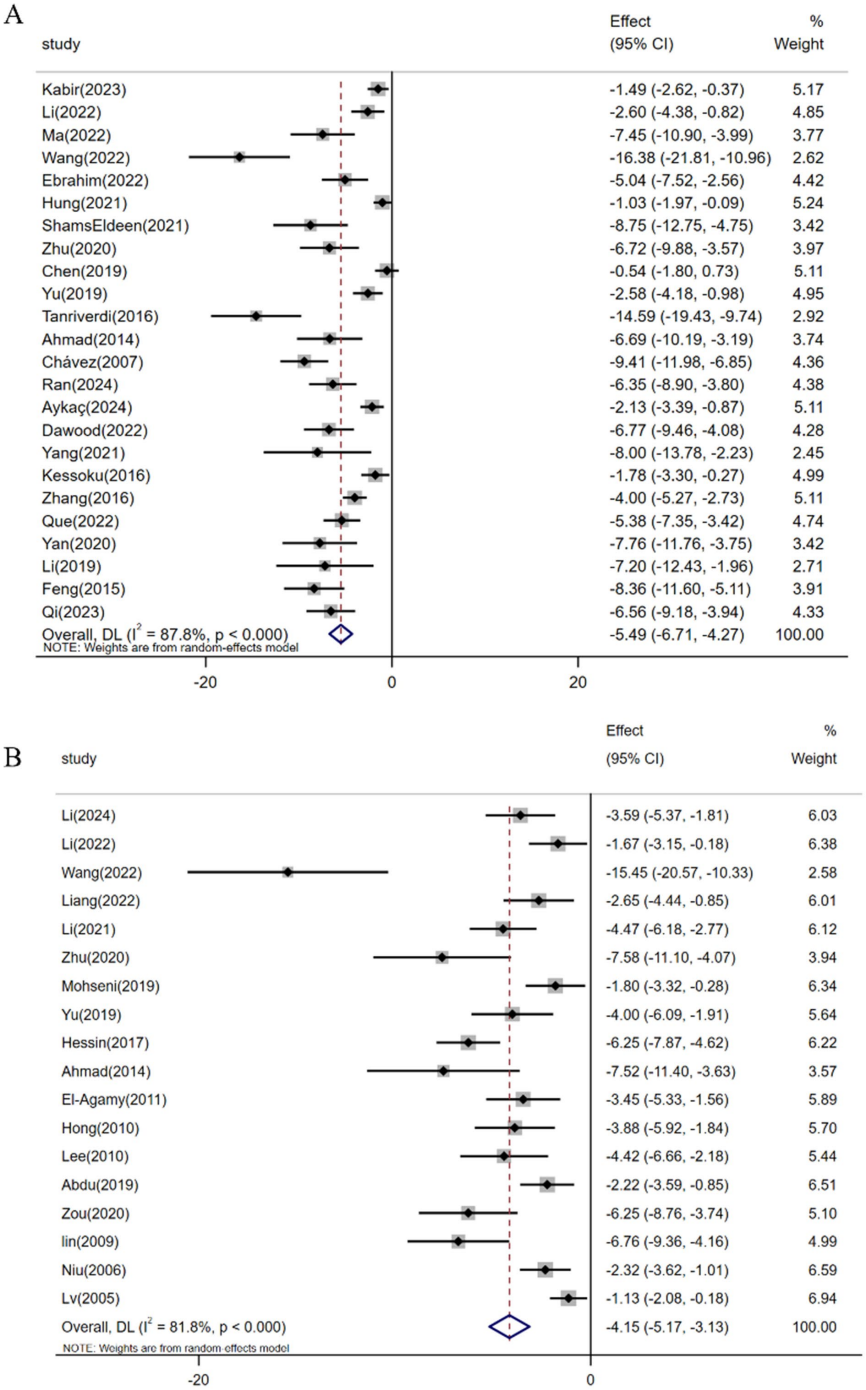
Forest plot: effect of resveratrol on **(A)** Hepatic collagen deposition and **(B)** HYP.

Among the 37 studies evaluating resveratrol’s antifibrotic effects, a meta-analysis of 24 investigations (*n* = 358) demonstrated a significant reduction in hepatic collagen deposition under pathological conditions [SMD: -5.49 (95% CI: −6.71, −4.27), *p* < 0.001; heterogeneity: *I*^2^ = 87.8%, *p* < 0.001; Figure 4A]. Hyp, a non-essential amino acid serving as a collagen-specific biomarker, reflects hepatic collagen synthesis status (70). Pooled analysis of 18 studies (*n* = 273) revealed resveratrol’s efficacy in reducing Hyp levels and ameliorating fibrotic progression in animal models [SMD: -4.15 (95% CI: −5.17, −3.13), *p* < 0.001; heterogeneity: *I*^2^ = 81.8%, *p* < 0.001; Figure 4B].

#### 3.4.2 Secondary outcomes

##### 3.4.2.1 Liver fibrosis-related biomarkers

**Figure 5:**
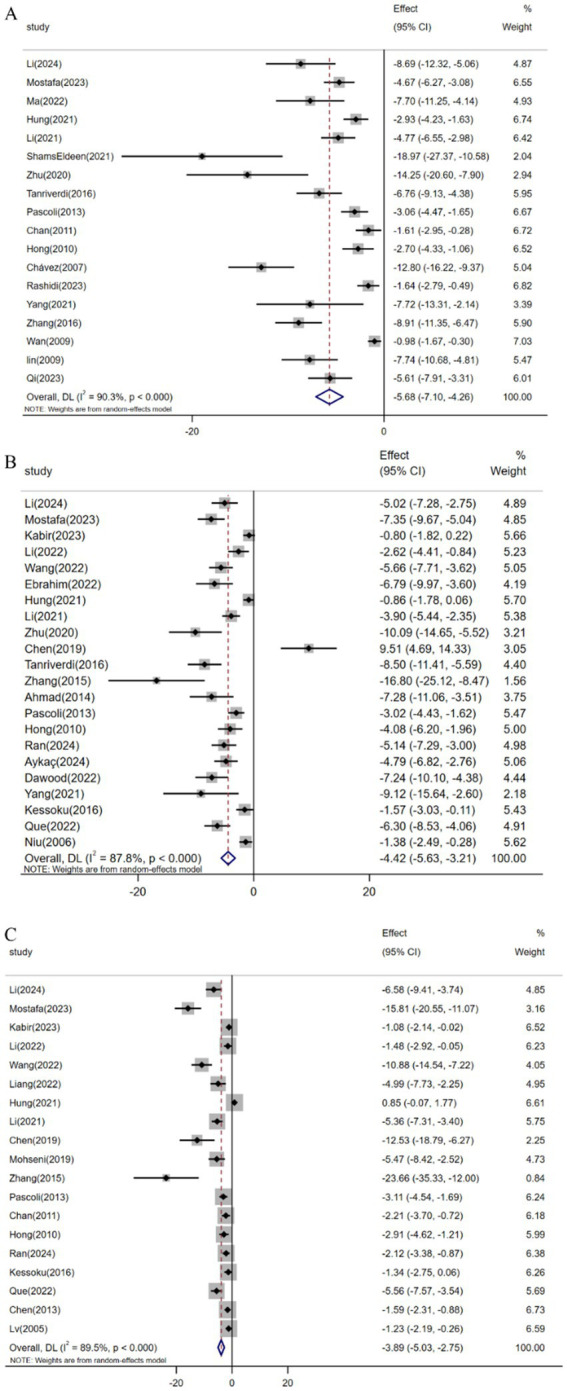
Forest plot: effect of resveratrol on **(A)** TGF-*β*, **(B)** *α*-SMA, **(C)** Col1α1.

Pooled analysis of 18 studies (*n* = 329) demonstrated resveratrol significantly suppressed TGF-*β* expression versus controls [SMD: -5.68 (95% CI: −7.10, −4.26), *p* < 0.001; *I*^2^ = 90.3%, *p* < 0.001; Figure 5A]. Similarly, meta-analysis of 22 studies (*n* = 328) revealed reduced *α*-SMA levels in resveratrol-treated groups [SMD: -4.42 (95% CI: −5.63, −3.21), *p* < 0.001; *I*^2^ = 87.8%, *p* < 0.001; Figure 5B]. Furthermore, analysis of 19 trials (*n* = 312) confirmed attenuated Col1α1 expression following resveratrol intervention [SMD: -3.89 (95% CI: −5.03, −2.75), *p* < 0.001; *I*^2^ = 89.5%, *p* < 0.001; Figure 5C].

##### 3.4.2.2 Liver function

**Figure 6:**
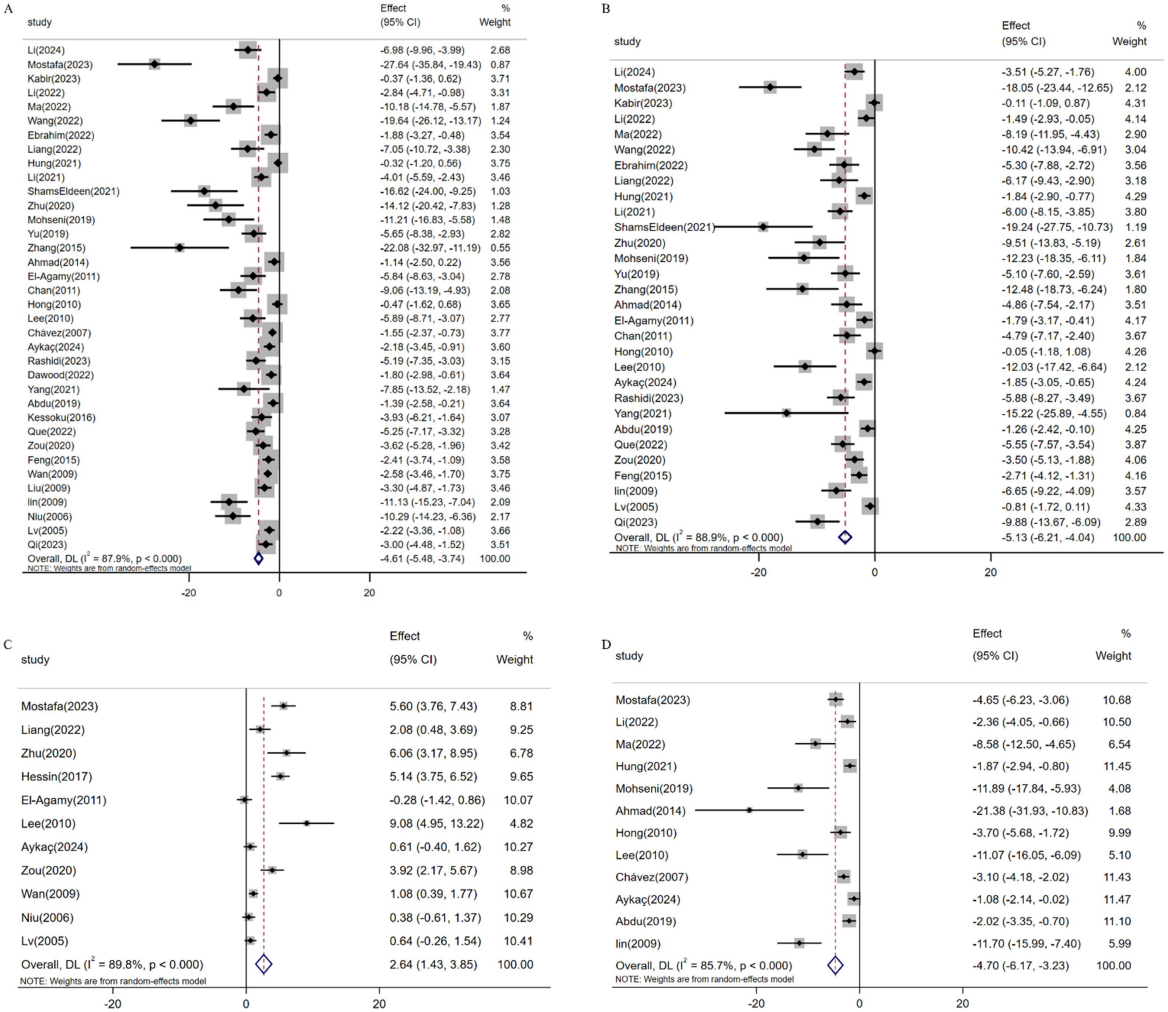
Forest plot: effect of resveratrol on **(A)** ALT, **(B)** AST, **(C)** ALB, **(D)** ALP.

Meta-analysis of 36 studies (*n* = 555) demonstrated resveratrol significantly reduced ALT levels versus controls [SMD: -4.61 (95% CI: −5.48, −3.74), *p* < 0.001; *I*^2^ = 87.9%; Figure 6A]. Similarly, pooled data from 30 studies (*n* = 429) revealed suppressed AST expression [SMD: -5.13 (95% CI: −6.21, −4.04; Figure 6B), *p* < 0.001; *I*^2^ = 88.9%]. Conversely, analysis of 11 studies (*n* = 212) confirmed elevated ALB levels following resveratrol treatment [SMD: 2.64 (95% CI: 1.43, 3.85), *p* < 0.001; I^2^ = 89.8%; Figure 6C]. Additionally, 12 studies (*n* = 187) showed reduced ALP activity [SMD: -4.70 (95% CI: −6.17, −3.23), *p* < 0.001; *I*^2^ = 85.7%; Figure 6D].

##### 3.4.2.3 Oxidative stress

**Figure 7:**
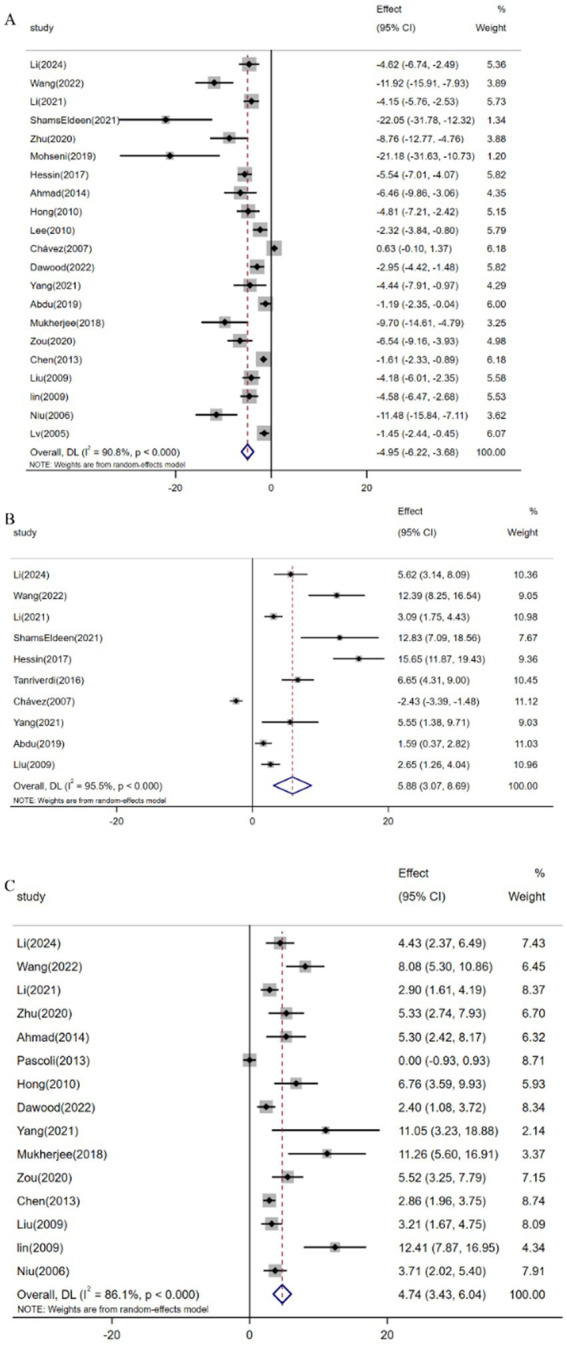
Forest plot: effect of resveratrol on **(A)** MDA, **(B)** GSH, **(C)** SOD.

Pooled analysis of 21 studies (*n* = 359) demonstrated resveratrol significantly reduced MDA levels versus controls [SMD: -4.95 (95% CI: −6.22, −3.68), *p* < 0.001; *I*^2^ = 90.8%; Figure 7A]. Similarly, meta-analysis of 10 studies (*n* = 188) revealed elevated GSH expression following resveratrol intervention [SMD: 5.88 (95% CI: 3.07, 8.69), *p* < 0.001; *I*^2^ = 95.5%; Figure 7B]. Furthermore, analysis of 15 trials (*n* = 243) confirmed increased SOD activity [SMD: 4.74 (95% CI: 3.43, 6.04), *p* < 0.001; *I*^2^ = 86.1%; Figure 7C].

##### 3.4.2.4 Inflammation

**Figure 8:**
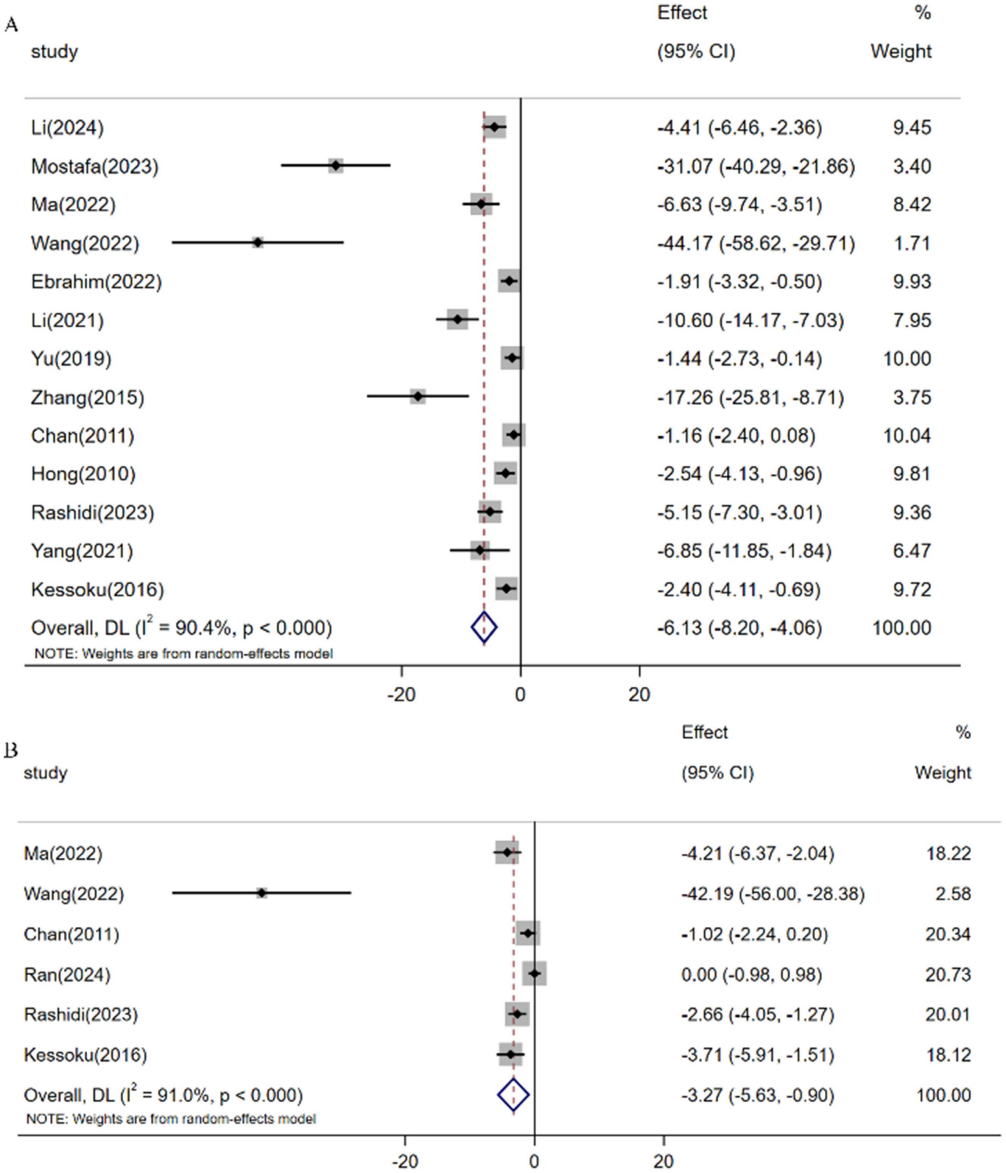
Forest plot: effect of resveratrol on **(A)** TNF-α and **(B)** IL-6.

Meta-analysis of 13 studies (*n* = 180) demonstrated resveratrol significantly reduced TNF-*α* levels versus controls [SMD: -6.13 (95% CI: −8.20, −4.07), *p* < 0.001; *I*^2^ = 90.4%; Figure 8A]. Similarly, pooled analysis of 6 trials (*n* = 86) revealed attenuated IL-6 expression following resveratrol intervention [SMD: -3.27 (95% CI: −5.63, −0.90), *p* < 0.001; *I*^2^ = 91.0%; Figure 8B].

##### 3.4.2.5 Extracellular matrix

**Figure 9:**
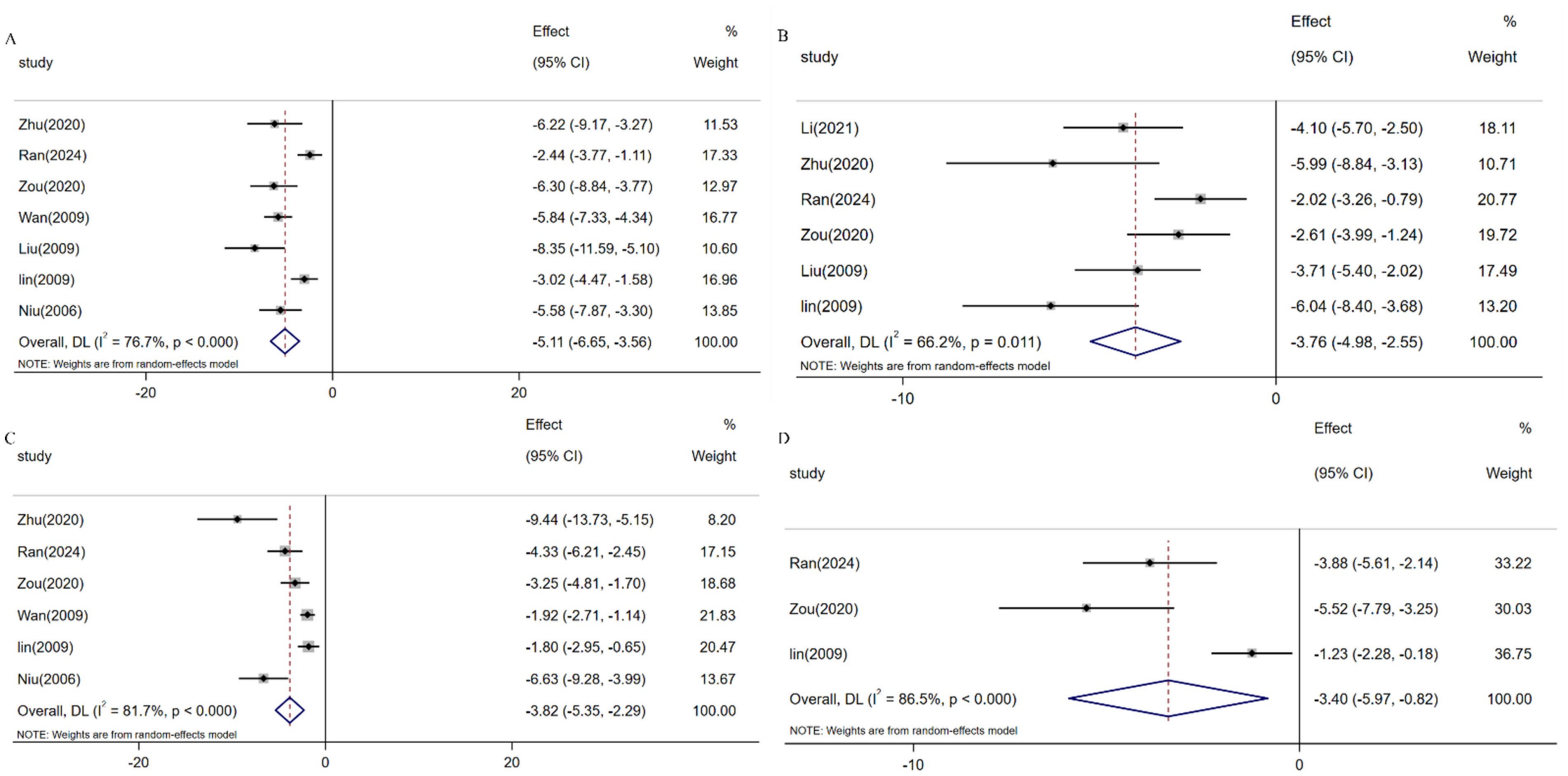
Forest plot: effect of resveratrol on **(A)** HA, **(B)** LN, **(C)** PIINP, and **(D)** COL-IV.

Serum biomarkers including HA, LN, PIINP, and COL-IV serve as critical indicators for assessing hepatic inflammatory activity and fibrotic progression, with elevated levels correlating with ECM deposition and hepatocyte injury (19782946). Meta-analysis of 7 studies (*n* = 131) demonstrated resveratrol significantly reduced HA expression versus controls [SMD: -5.11 (95% CI: −6.65, −3.56), *p* < 0.001; *I*^2^ = 76.7%; Figure 9A]. Similarly, pooled analysis of 6 trials (n = 97) revealed attenuated LN levels [SMD: -3.77 (95% CI: −4.98, −2.55), *p* < 0.001; *I*^2^ = 66.2%; Figure 9B], while 6 studies (*n* = 115) showed suppressed PIINP expression [SMD: -3.82 (95% CI: −5.35, −2.29), *p* < 0.001; *I*^2^ = 81.7%; Figure 9C]. Additionally, 3 studies (*n* = 49) confirmed reduced COL-IV levels following resveratrol intervention [SMD: -3.40 (95% CI: −5.97, −0.82), *p* < 0.001; *I*^2^ = 86.5%; Figure 9D].

### 3.5 Sensitivity analysis

Sensitivity analysis excluded through sequential research indicates that despite high heterogeneity, the estimated effects of all outcome measures were stable. The exclusion of Chávez (51) and Hung et al. (64) yielded marginal variations in collagen deposition effect sizes (minimum: −3.04, 95% CI: −3.43 to −2.65; maximum: −3.64, 95% CI: −4.07 to −3.21). Similarly, removing Hessin et al. (47) and Lv (2005) revealed comparable HYP effect size ranges (minimum: −2.93, 95% CI: −3.36 to −2.51; maximum: −3.61, 95% CI: −4.07 to −3.16). The results of secondary outcomes still have robustness (Supplementary Table 4).

### 3.6 Subgroup analysis

To address substantial between-study heterogeneity, stratified subgroup analyses of collagen deposition and HYP levels were performed across four covariates: disease induction methods, animal species, administration routes, and dosing regimens. The analysis revealed disease modeling approaches, animal species selection, and treatment duration as key contributors to collagen deposition heterogeneity. For HYP variability, primary sources included disease induction protocols, administration methods, and dosage parameters. Comprehensive stratification data are presented in the attached Supplementary Table 3.

### 3.7 Publication bias

**Figure 10:**
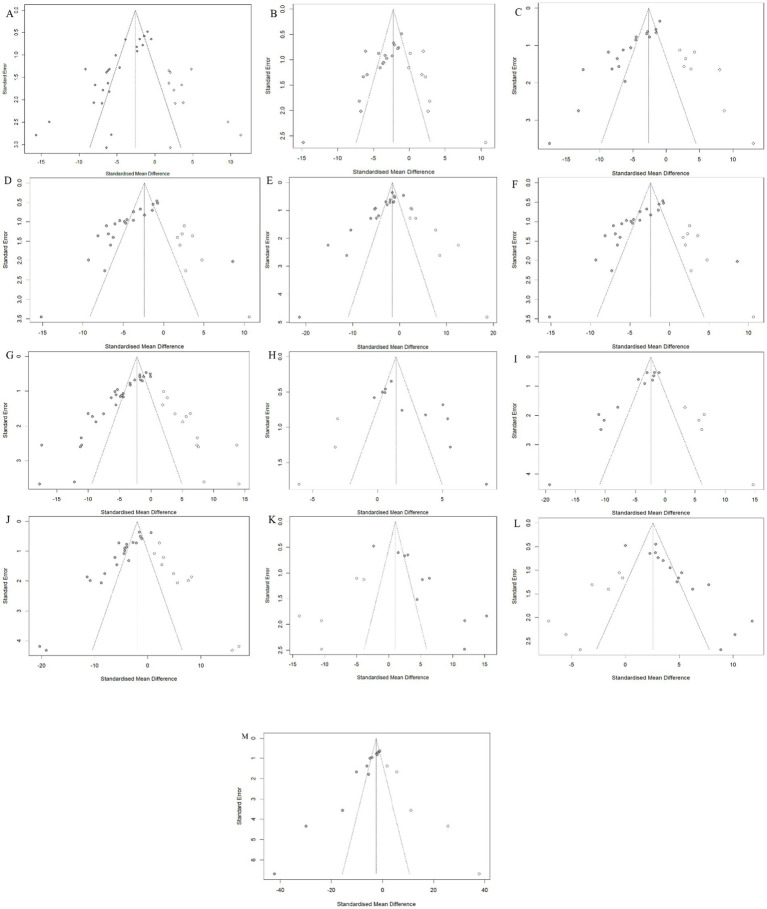
Trim-and-fill analysis for **(A)** Hepatic collagen deposition, **(B)** HYP, **(C)** TGF-β, **(D)** α-SMA, **(E)** Col1α1, **(F)** ALT, **(G)** AST, **(H)** ALB, **(I)** ALP, **(J)** MDA, **(K)** GSH, **(L)** SOD and **(M)** TNF-α.

In the presence of sufficient data, Egger and Begg were used to assess publication bias, and the results showed that all outcome measures included in the analysis were at risk of publication bias (Supplementary Figure 1). To address potential missing research effects, sensitivity analysis conducted through the pruning and filling methods showed that unpublished data had no significant impact on the combined effect estimation of degree of liver fibrosis, HYP, *α*-SMA, ALT, AST, and SOD, and the research results were robust (Table 2; Figure 10). However, unpublished data had a significant impact on the combined effect estimation of TGF -*β*, Col1α1, ALB, ALP, MDA, GSH, and TNF-α, and the results obtained from the study were not robust.

**Table 2:** The results from the trim-and-fill analysis.

	Before trim and fill			After trim and fill		
Parameter	*p* value	SMD	No. studies	*P* value	SMD	No. studies
Collagen deposition	*p* < 0.05	−5.49	24	*p* < 0.05	−2.58	35
HYP	*p* < 0.05	−4.15	18	*p* < 0.05	−2.24	26
TGF-β	*p* < 0.05	−5.68	18	*p* = 0.06	−2.63	26
α-SMA	*p* < 0.05	−4.42	22	*p* < 0.05	−2.39	30
Col1α1	*p* < 0.05	−3.89	19	*p* = 0.29	−1.54	27
ALT	*p* < 0.05	−4.61	36	*p* < 0.05	−2.39	30
AST	*p* < 0.05	−5.13	30	*p* < 0.05	−2.33	43
ALB	*p* < 0.05	2.64	11	*p* = 0.18	1.43	14
ALP	*p* < 0.05	−4.70	12	*p* = 0.21	−2.40	17
MDA	*p* < 0.05	−4.95	21	*p* = 0.16	−1.96	31
GSH	*p* < 0.05	5.88	10	*p* = 0.64	1.06	15
SOD	*p* < 0.05	4.74	15	*p* < 0.05	2.54	22
TNF-α	*p* < 0.05	−6.13	13	*p* = 0.54	−2.52	18

## 4 Discussion

### 4.1 Effectiveness and evidence summary

This meta-analysis synthesizing 46 preclinical studies demonstrates resveratrol’s therapeutic potential against hepatic fibrosis by attenuating collagen deposition and HYP accumulation, suppressing fibrogenic markers (TGF-β, α-SMA, Col1α1), and modulating ECM components (HA, LN, PIIINP, COL-IV). Hepatoprotective effects were evidenced by reduced ALT/AST/ALP levels and elevated albumin expression. Notwithstanding substantial heterogeneity observed in primary outcomes (collagen deposition: I^2^ = 87.8%; HYP: I^2^ = 81.8%), sensitivity analyses confirmed result stability. Stratified subgroup analyses identified disease induction methods, animal species, administration routes, and dosage regimens as key heterogeneity contributors. Potential publication bias was identified in all results, the robustness of secondary outcome measures decreased after adjusting for pruning and padding, indicating that the bias may have been exaggerated.

### 4.2 Potential mechanism

The activation of HSC constitutes the central pathological mechanism underlying LF (71, 72). Activated HSCs generate excessive reactive oxygen species (ROS) (73), inducing persistent oxidative stress that compromises cellular membrane integrity and organelle architecture, ultimately driving hepatocyte injury, necrosis, and apoptosis (74). This cyclical process perpetuates HSC activation and fibrotic progression. Mechanistically, MDA, a terminal lipid peroxidation byproduct, serves as a dual biomarker of oxidative damage severity and hepatocyte injury (75, 76). SOD, the primary oxygen radical scavenger, becomes depleted under oxidative assault, while its restoration inhibits MDA-mediated free radical generation (77). GSH depletion exacerbates mitochondrial ROS leakage, triggering apoptotic cascades and profibrotic factor release (78). Resveratrol shows anti-fibrotic potential in preclinical studies via MDA reduction and enhanced SOD/GSH defenses, indicating oxidative stress mitigation.

Fibrogenesis critically depends on inflammatory dysregulation, where early repair-to-pathology transition follows pro−/anti-inflammatory imbalance (79). Chronic inflammation, whether driven by steatosis-induced lipotoxicity or ROS-mediated oxidative stress (80), activates proapoptotic pathways and inflammatory cascades, perpetuating HSC activation via phagocytosis of cellular debris (81–83). Interleukin is a key link in immune regulation. Among them, IL-6 plays a role in promoting fibrosis, and IL-22, IL-24 exerts anti-fibrotic effects (10). TNF-*α* can regulate the expression of matrix metalloproteinase (MMP) -9, thereby promoting LF (84). Oxidative stress can also activate NF-κB, promote the release of inflammatory factors such as TNF-α and IL-6, exacerbate inflammatory reactions, and lead to fibrosis (85). In addition, inflammation can induce epithelial-mesenchymal transition in liver cells which was associated with reduced TGF-*β* expression. TGF-β is the initial signal for activation and transformation of quiescent HSC and plays an important role in the occurrence and development of LF (86–88). Studies have shown that inhibiting the TGF-β/SMAD signaling pathway can alleviate the progression of LF (89). Resveratrol can downregulate the expression of TNF-α, IL-6, and TGF-β. Therefore, we speculate that resveratrol may delay the progression of LF by inhibiting inflammatory responses and the TGF-β/SMAD signaling pathway.

### 4.3 Heterogeneity of methodology and animal models

The substantial heterogeneity observed in this study primarily stems from differences in experimental design and limitations of animal models. Chemically induced models (e.g., CCl₄/TAA) induce rapid fibrosis through acute liver injury, whereas metabolic models (high-fat diet/choline deficiency) replicate the progressive mechanism of NAFLD. Consequently, resveratrol’s inhibitory effect on TGF-β is markedly weaker in metabolic models compared to chemical models. Meanwhile, the essential differences between the CYP1B1 metabolism of resveratrol in rodents and the human CYP1A2 pathway, as well as the lack of core comorbidity features of human liver fibrosis in existing models (such as insulin resistance and gut microbiota disorders), further weaken the clinical extrapolation of the results.

### 4.4 Clinical translational disorders

The 46 preclinical studies included in this meta-analysis lacked dose-ranging toxicity evaluations and standardized documentation of organ-specific adverse reactions (e.g., weight loss, multi-organ injury, or abnormal mortality). Although doses varied significantly (10–500 mg/kg), only 17.4% (8/46) monitored biochemical parameters beyond baseline liver function (ALT/AST), and none provided histopathological assessment of extrahepatic organs (e.g., kidneys, heart). This precludes assessment of whether resveratrol’s known pharmacological risks (e.g., CYP450 enzyme inhibition or estrogen receptor modulation) manifest at anti-fibrotic doses. Furthermore, the longest-duration study (36 weeks) evaluated efficacy endpoints exclusively, neglecting chronic exposure cumulative toxicity assessment. Consequently, neither the No Observed Adverse Effect Level (NOAEL) nor the safety margin required for regulatory dose translation could be established.

### 4.5 Optimization path for future research

Future research must mandate tiered dosing designs (encompassing 20–50 mg/kg human-equivalent and 2–5 therapeutic doses), pathological screening of core organs (liver/kidney/heart), and dynamic CYP450 activity monitoring to address critical gaps in systematic safety reporting. In the preclinical phase, humanized liver models (e.g., FRG mice) or organoid co-culture systems should integrate metabolic-inflammatory interactions, alongside establishing multi-etiological sequential injury models (e.g., HCV infection combined with high-fat diet). In addition, future research on the mechanism of action can focus on microRNAs to comprehensively elucidate the role of resveratrol in preventing and treating LF. Early clinical trials should prioritize liposomal formulations like ResVida®, conducting Phase I maximum tolerated dose studies focused on CYP450/hormonal disturbances while quantifying target engagement (e.g., p-Smad2/3 inhibition rates) via Phase IIa liver biopsies. Ultimately, a precision treatment framework should be developed using validated biomarkers (plasma miR-29a, CK-18) for cohort stratification, coupled with antioxidant-synergistic combination regimens such as obeticholic acid or selonsertib.

## 5 Conclusion

Preclinical evidence demonstrates resveratrol’s capacity to attenuate hepatic fibrogenesis and restore hepatic functional markers in animal models. Mechanistically, the observed therapeutic effects coincide with concurrent improvements in both inflammatory markers and oxidative stress parameters. Despite resveratrol’s anti-fibrotic potential in preclinical studies, clinical validation remains essential for therapeutic translation.

## Data Availability

The original contributions presented in the study are included in the article/Supplementary material, further inquiries can be directed to the corresponding author/s.

## Footnotes

^1^ https://apps.automeris.io/wpd4/

## Glossary

HYP: hydroxyproline
ALT: alanine aminotransferase
AST: aspartate aminotransferase
*α*-SMA: α-smooth muscle actin
TGF-*β*: transforming growth factor-β
Col1α1: collagen type I alpha 1
MDA: malondialdehyde
SOD: superoxide dismutase
GSH: glutathione
IL-6: interleukin-6
TNF-α: tumor necrosis factor-α
HA: hyaluronic acid
LN: laminin
CIV: type IV collagen
PIIINP: type III procollagen N-terminal peptide
ALP: alkaline phosphatase
ALB: albumin
NF-Κb: nuclear factor kappa-B
ECM: extracellular matrix
ROS: reactive oxygen species
LF: Liver fibrosis
