# Supplementary material for: The efficacy of resveratrol in the treatment of liver fibrosis: a systematic review and meta-analysis of preclinical studies
Source: Front Nutr. 2025 Sep 19;12:1606603. doi: 10.3389/fnut.2025.1606603 (PMC12491981; doi:10.3389/fnut.2025.1606603)
Supplement: Supplementary file 1 [file Supplementary_file_1.docx]

**Supplementary materials**

**Supplementary Table 1**

Literature search strategy for resveratrol treatment of liver fibrosis

| PubMed Search Strategy | |
| --- | --- |
| #1 | Resveratrol [Mesh] |
| #2 | 3,4',5-Stilbenetriol [Title/Abstract] |
| #3 | 3,5,4'-Trihydroxystilbene [Title/Abstract] |
| #4 | 3,4',5-Trihydroxystilbene [Title/Abstract] |
| #5 | Trans-Resveratrol [Title/Abstract] |
| #6 | Trans Resveratrol [Title/Abstract] |
| #7 | Resveratrol-3-sulfate [Title/Abstract] |
| #8 | Resveratrol 3 sulfate [Title/Abstract] |
| #9 | SRT 501[Title/Abstract] |
| #10 | SRT-501 [Title/Abstract] |
| #11 | SRT501 [Title/Abstract] |
| #12 | Cis-Resveratrol [Title/Abstract] |
| #13 | Cis Resveratrol [Title/Abstract] |
| #14 | Resveratrol, (Z)- [Title/Abstract] |
| #15 | Trans-Resveratrol-3-O-sulfate [Title/Abstract] |
| #16 | Trans Resveratrol 3 O sulfate [Title/Abstract] |
| #17 | #1 OR #2 OR #3 OR #4 OR #5 OR #6 OR #7 OR #8 OR #9 OR #10 OR #11 OR #12 OR #13 OR #14 OR #15 OR #16 |
| #18 | Liver Cirrhosis [Mesh] |
| #19 | Cirrhosis liver [Title/Abstract] |
| #20 | Hepatic cirrhosis [Title/Abstract] |
| #21 | Cirrhosis hepatic [Title/Abstract] |
| #22 | Fibrosis liver [Title/Abstract] |
| #23 | Liver fibrosis [Title/Abstract] |
| #24 | #18 OR #19 OR #20 OR #21 OR #22 OR #23 |
| #25 | #17 AND #24 |
| Web of science search strategy | |
| #1 | (((((((((((((((TS=(Resveratrol)) OR TS=(3,4',5-Stilbenetriol)) OR TS=(3,5,4'-Trihydroxystilbene)) OR TS=(3,4',5-Trihydroxystilbene)) OR TS=(Trans-Resveratrol)) OR TS=(Trans Resveratrol)) OR TS=(Resveratrol-3-sulfate)) OR TS=(Resveratrol 3 sulfate)) OR TS=(SRT 501)) OR TS=(SRT-501)) OR TS=(SRT501)) OR TS=( Cis-Resveratrol)) OR TS=(Cis Resveratrol)) OR TS=(Resveratrol, (Z)-)) OR TS=(Trans-Resveratrol-3-O-sulfate)) OR TS=(Trans Resveratrol 3 O sulfate)) |
| #2 | (((((TS=(Liver Cirrhosis)) OR TS=(Cirrhosis liver)) OR TS=(Hepatic cirrhosis)) OR TS=(Cirrhosis hepatic)) OR TS=(Fibrosis liver)) OR TS=(Liver fibrosis)) |
| #3 | #1 AND #2 |
| Embase search strategy | |
| #1 | 'resveratrol'/exp |
| #2 | '3, 4`, 5 stilbenetriol' OR '3, 4`, 5 trihydroxystilbene' OR '5 (4 hydroxystyryl) benzene 1, 3 diol' OR 'srt 501' OR 'srt501' OR 'trans resveratrol' OR 'trans-resveratrol' OR 'resveratrol':ab, ti |
| #3 | #1 OR #2 |
| #4 | 'liver fibrosis'/exp |
| #5 | 'fibrosis, liver' OR 'fibrous hepatic disease' OR 'hepatic fibrosis' OR 'liver periportal fibrosis' OR 'periportal fibrosis' OR 'liver fibrosis':ab, ti |
| #6 | #4 OR #5 |
| #7 | #3 AND #6 |
| CBM search strategy | |
| #1 | Resveratrol [Mesh] |
| #2 | Trans Resveratrol |
| #3 | Resveratrol 3 sulfate |
| #4 | Cis Resveratrol |
| #5 | Trans Resveratrol 3 O sulfate |
| #6 | #1 OR #2 OR #3 OR #4 OR #5 |
| #7 | Liver Cirrhosis [Mesh] |
| #8 | Liver fibrosis [Mesh] |
| #9 | #7 OR #8 |
| #10 | #6 AND #9 |
| CNKI search strategy | |
| #1 | Resveratrol (subjects) OR Trans Resveratrol (subjects) OR Resveratrol 3 sulfate (subjects) OR Trans Resveratrol 3 O sulfate (subjects)OR Cis Resveratrol (subjects) |
| #2 | Liver Cirrhosis (subjects) OR Liver fibrosis (subjects) |
| #3 | #1 AND #2 |
| WF search strategy | |
| #1 | Subjects:(“Resveratrol” OR “Trans Resveratrol” OR “Resveratrol 3 sulfate” OR “Trans Resveratrol 3 O sulfate” OR “Cis Resveratrol”) AND subjects(“Liver Cirrhosis” OR “Liver fibrosis”) |
| VIP search strategy | |
| #1 | K=(Resveratrol OR Trans Resveratrol OR Resveratrol 3 sulfate OR Trans Resveratrol 3 O sulfate OR Cis Resveratrol) AND K=( Liver Cirrhosis OR Liver fibrosis) |

**Supplementary Table 2** Risk of Bias Summary

| Author(year) | A | B | C | D | E | F | G | H | I | J | Total |
| --- | --- | --- | --- | --- | --- | --- | --- | --- | --- | --- | --- |
| Li (2024) | + | + | ? | ? | ? | + | ? | + | + | + | 6 |
| Mostafa(2023) | + | ? | ? | + | ? | + | ? | + | + | + | 6 |
| Kabir(2023) | ? | + | ? | ? | ? | + | ? | + | + | + | 5 |
| Li(2022) | + | + | ? | ? | ? | + | ? | + | + | + | 6 |
| Ma(2022) | ? | + | ? | ? | ? | + | + | + | + | + | 6 |
| Wang(2022) | + | + | ? | + | ? | + | ? | + | + | + | 6 |
| Ebrahim(2022) | ? | + | ? | ? | ? | ? | ? | + | + | + | 4 |
| Liang(2022) | ? | + | ? | ? | ? | ? | ? | + | + | + | 4 |
| Hung(2021) | + | + | ? | + | ? | ? | ? | + | + | + | 6 |
| Li(2021) | + | + | ? | + | ? | + | ? | + | + | + | 7 |
| ShamsEldeen(2021) | + | + | ? | + | ? | + | ? | + | + | + | 7 |
| Zhu(2020) | + | + | ? | + | ? | + | ? | + | + | + | 7 |
| Chen(2019) | + | + | ? | ? | ? | + | ? | + | + | + | 6 |
| Mohseni(2019) | + | + | ? | ? | ? | + | ? | + | + | + | 6 |
| Yu(2019) | ? | + | ? | + | ? | ? | ? | + | + | + | 5 |
| Hessin(2017) | + | + | ? | + | ? | ? | ? | + | + | + | 6 |
| Tanriverdi(2016) | ? | + | ? | ? | ? | + | + | + | + | + | 6 |
| Zhang(2015) | + | + | ? | + | ? | ? | ? | + | + | + | 6 |
| Ahmad(2014) | ? | + | ? | + | ? | ? | ? | + | + | + | 5 |
| Pascoli(2013) | + | + | ? | + | ? | + | + | + | + | + | 8 |
| El-Agamy(2011) | ? | + | ? | + | ? | + | + | + | + | + | 7 |
| Chan(2011) | + | + | ? | ? | ? | ? | ? | + | + | + | 5 |
| Hong(2010) | ? | + | + | ? | ? | ? | ? | + | + | + | 5 |
| Lee(2010) | ? | + | + | ? | ? | ? | ? | + | + | + | 5 |
| Chávez(2007) | ? | + | ? | ? | ? | ? | ? | + | + | + | 4 |
| Ran(2024) | ? | + | ? | ? | ? | ? | ? | + | + | + | 4 |
| Aykaç(2024) | + | + | ? | + | ? | + | + | + | + | + | 8 |
| Rashidi(2023) | ? | + | ? | ? | ? | + | + | + | + | + | 6 |
| Dawood(2022) | ? | + | ? | ? | ? | ? | ? | + | + | + | 4 |
| Yang(2021) | + | + | ? | + | ? | + | + | + | + | + | 8 |
| Abdu(2019) | + | + | ? | + | ? | ? | ? | + | + | + | 6 |
| Mukherjee(2018) | + | + | ? | + | ? | ? | ? | + | + | + | 6 |
| Kessoku(2016) | + | + | ? | + | ? | + | + | + | + | + | 8 |
| Zhang(2016) | + | + | ? | ? | ? | ? | ? | + | + | + | 5 |
| Que(2022) | + | + | ? | + | ? | ? | ? | + | + | + | 6 |
| Zou(2020) | + | + | ? | + | ? | ? | ? | + | + | + | 6 |
| Yan(2020) | + | + | ? | + | ? | ? | ? | + | + | + | 6 |
| Li(2019) | + | + | ? | ? | ? | ? | ? | + | + | + | 5 |
| Feng(2015) | + | + | ? | ? | ? | ? | ? | + | + | + | 5 |
| Chen(2013) | + | + | ? | ? | ? | ? | ? | + | + | + | 5 |
| Wan(2009) | + | + | ? | ? | ? | ? | ? | + | + | + | 5 |
| Liu(2009) | + | + | ? | ? | ? | ? | ? | + | + | + | 5 |
| lin(2009) | + | + | ? | ? | ? | ? | ? | + | + | + | 5 |
| Niu(2006) | + | + | ? | + | ? | ? | ? | + | + | + | 6 |
| Lv(2005) | ? | + | ? | + | ? | ? | ? | + | + | + | 5 |
| Qi(2023) | + | + | ? | + | ? | ? | ? | + | + | + | 6 |

1. Sequence generation. (B) Baseline characteristics. (C) Allocation concealment. (D) Random housing. (E) Blinding of experimentalists. (F) Random outcome assessment. (G) Blinding of outcome assessors. (H) Incomplete outcome data. (I) Selective outcome reporting. (J) Other sources of bias. +: indicates low risk; -: indicates high risk; ?: indicates unclear risk.

**Supplementary Table 3** The subgroup analyses of degree of liver fibrosis and hydroxyproline.

| **Outcome** | **Subgroup** |  | **No. studies** | **SMD [95% CI]** | ***I*^2^** |
| --- | --- | --- | --- | --- | --- |
| Degree of liver fibrosis | Modeling method | **CCL4** | 13 | -5.88[-7.70, -4.06] | **88.3** |
|  |  | high-fat diet | 2 | -1.59 [-2.50, -0.69] | **0%** |
|  |  | Thioacetamide | 2 | -5.83[-7.66, -4.01] | **0%** |
|  |  | **N0-nitrosodimethylamine** | 2 | -6.61[-8.70, -4.51] | **0%** |
|  |  | Schistosoma | 2 | -2.27[-5.66,1.13] | **93.0%** |
|  |  | **Other** | 3 | -10.03[-15.24,-4.83] | **87.8%** |
|  | Species | **Sprague-Dawley rats** | 6 | -5.87 [-8.28,-3.46] | **78.9%** |
|  |  | **Wistar rats** | 2 | -9.22[-11.38,-7.06] | **0%** |
|  |  | **C57BL/6J mice** | 5 | -3.85[-5.76,-1.95] | **85.7%** |
|  |  | **Balb/c** | 5 | -3.23[-5.27,-1.18] | **77.5%** |
|  |  | Agouti rats | 4 | -7.76[-10.97,-4.56] | **74.7%** |
|  |  | **Other** | 2 | -8.46[-23.50,6.58] | **96.7%** |
|  | Administration method | **Intraperitoneal injection** | 9 | -5.51[-7.69,-3.33] | **88.0%** |
|  |  | **Gavage or peros** | 15 | -5.55[-7.12,-3.99] | **88.5%** |
|  | Administered dose | 1-10 mg/kg | 4 | -7.92[-13.14,-2.70] | **93.4%** |
|  |  | 11-20mg/kg | 5 | -4.56[-6.33,-2.79] | **75.3%** |
|  |  | 21-30mg/kg | 6 | -5.25[-7.91,-2.60] | **89.4%** |
|  |  | 31-100 mg/kg | 2 | -11.17[-20.78,-1.56] | **90.2%** |
|  |  | 101-200mg/kg | 2 | -3.89[-9.00,1.22] | **89.3%** |
|  |  | 201-400mg/kg | 5 | -4.33[-7.11,-1.55] | **81.4%** |
|  | **Administration time** | 3 weeks | 3 | -5.09[-10.69,0.51] | **91.3%** |
|  |  | 4 weeks | 5 | -3.52[-5.10,-1.94] | **71.1%** |
|  |  | 5 weeks | 2 | -7.55[-10.73,-4.37] | **0%** |
|  |  | 6-7 weeks | 4 | -9.81[-14.90,-4.72] | **91.4%** |
|  |  | **8-10** weeks | 6 | -6.26[-9.78,-2.74] | **92.2%** |
|  |  | ＞10 weeks | 4 | -3.89[-6.20,-1.59] | **85.1%** |
| HYP | Modeling method | **CCL4** | 8 | -2.95[-4.13,-1.78] | **74.8%** |
|  |  | Thioacetamide | 1 | -6.25[-7.87,-4.63] | **-** |
|  |  | **N0-nitrosodimethylamine** | 4 | -3.97 [-5.77,-2.18] | **63.2%** |
|  |  | Schistosoma | 1 | -3.45[-5.33,-1.56] | **-** |
|  |  | **Other** | 4 | -6.68[-9.81,-3.55] | **85.4%** |
|  | Species | **Sprague-Dawley rats** | 5 | -4.54[-6.32,-2.77] | **70.1%** |
|  |  | **Wistar rats** | 4 | -2.63[-4.34,-0.92] | **81.5%** |
|  |  | **C57BL/6J mice** | 1 | -2.65[-4.44,-0.85] | **-** |
|  |  | **Balb/c** | 4 | -3.63[-5.51,-1.75] | **68.0%** |
|  |  | Agouti rats | 1 | -6.25[-7.87,-4.63] | **-** |
|  |  | **Other** | 3 | -6.88[-11.03,-2.73] | **89.2%** |
|  | Administration method | **Intraperitoneal injection** | 3 | -4.17[-6.40,-1.94] | **60.8%** |
|  |  | **Gavage or peros** | 14 | -4.00[-5.17,-2.83] | **84.0%** |
|  |  | **Other** | 1 | -6.25[-8.76,-3.74] | **-** |
|  | Administered dose | 1-10 mg/kg | 3 | -3.82[-6.31,-1.33] | **78.6%** |
|  |  | 11-20mg/kg | 3 | -3.12[-4.38,-1.86] | **33.2%** |
|  |  | 21-30mg/kg | 3 | -4.81[-8.24,-1.38] | **90.5%** |
|  |  | 31-100 mg/kg | 4 | -4.55[-7.61,-1.49] | **90.9%** |
|  |  | 101-200mg/kg | 2 | -4.69[-9.82,0.45] | **86.8%** |
|  |  | 201-400mg/kg | 3 | -4.69[-6.15,-3.23] | **20.1%** |
|  | **Administration time** | 3 weeks | 3 | -2.95[-3.96,-1.93] | **6.6%** |
|  |  | 4 weeks | 8 | -4.74[-6.31,-3.17] | **76.0%** |
|  |  | 6-7 weeks | 2 | -8.06[-22.09,5.97] | **96.6%** |
|  |  | **8-20** weeks | 3 | -3.36[-5.69,-1.02] | **81.9%** |
|  |  | ＞10 weeks | 2 | -4.05[-5.28,-2.82] | **0%** |

**Supplementary Table 4** Sensitivity analysis of secondary outcome measures

|  | **Exclude study** | **Maximum effect quantity** | **Exclude study** | **Minimum effect quantity** |
| --- | --- | --- | --- | --- |
| TGF-β | Rashidi(2023) | -6.04(-7.58, -4.50) | Chávez(2007) | -5.20(-6.54, -3.86) |
| α-SMA | Chen(2019) | -4.79(-5.94, -3.64) | Tanriverdi(2016) | -4.21(-5.41, -3.01) |
| Col1α1 | Chen(2013) | -4.20(-5.51, -2.90) | Mostafa(2023) | -3.39(-4.44, -2.33) |
| ALT | Chávez(2007) | -4.81(-5.73, -3.89) | Mostafa(2023) | -4.30(-5.12, -3.48) |
| AST | Lv(2005) | -5.37(-6.51, -4.23) | Mostafa(2023) | -4.76(-5.79, -3.73) |
| ALB | El-Agamy(2011) | 2.97(1.68, 4.26) | Hessin(2017) | 2.27(1.15,3.40) |
| ALP | Hung(2021) | -5.25(-6.96, -3.55) | lin(2009) | -4.07(-5.44, -2.70) |
| MDA | Chen(2013) | -5.32(-6.77, -3.87) | Wang(2022) | -4.60(-5.84, -3.36) |
| GSH | Chávez(2007) | 6.73(4.30, 9.17) | Hessin(2017) | 4.76(2.16,7.36) |
| SOD | Chen(2013) | 5.05(3.52, 6.58) | lin(2009) | 4.31(3.08, 5.55) |
| TNF-α | Chan(2011) | -6.94(-9.28, -4.60) | Mostafa(2023) | -4.92(-6.72, -3.12) |

**Supplementary Figure 1** The results of the publication bias

**
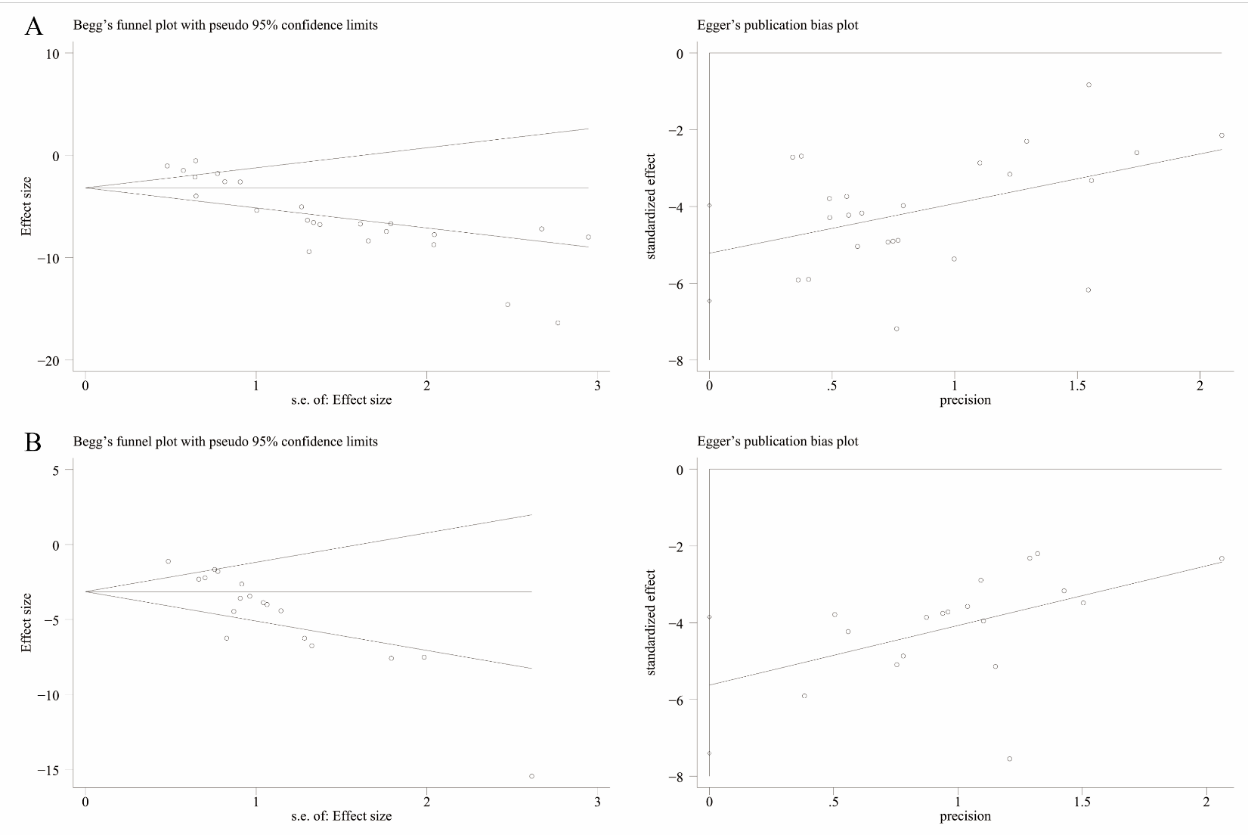
**

**
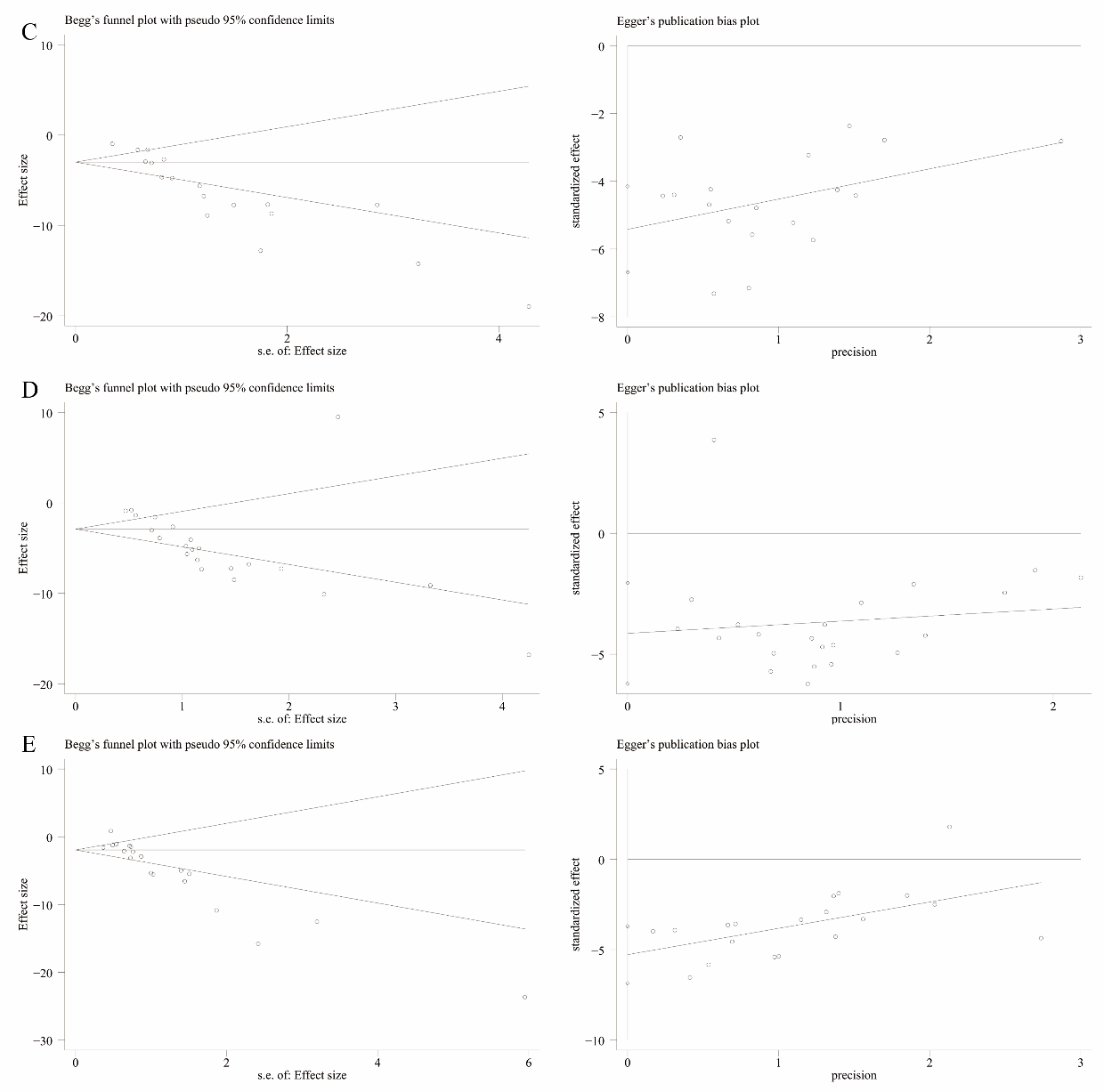
**

**
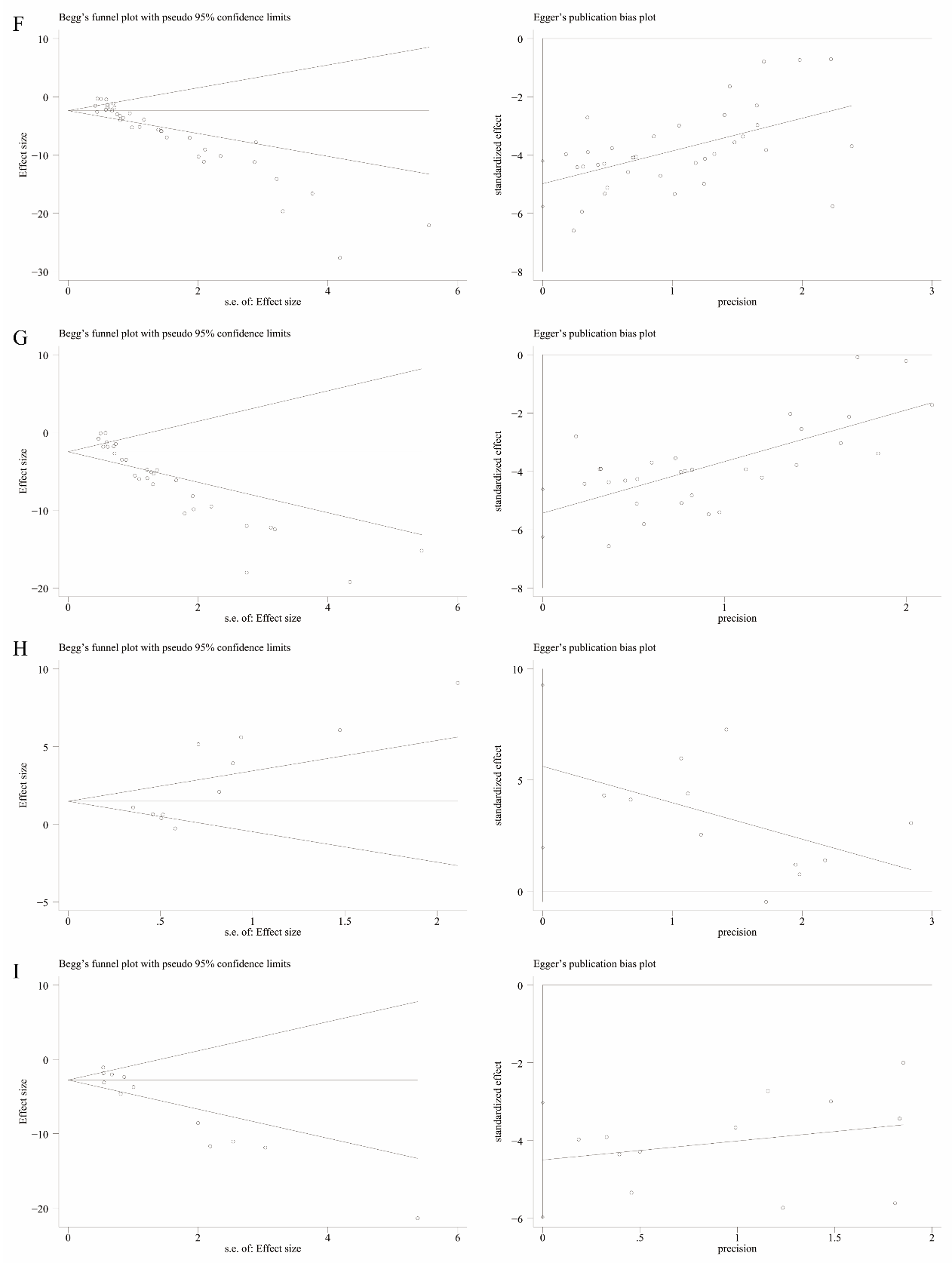
**

**
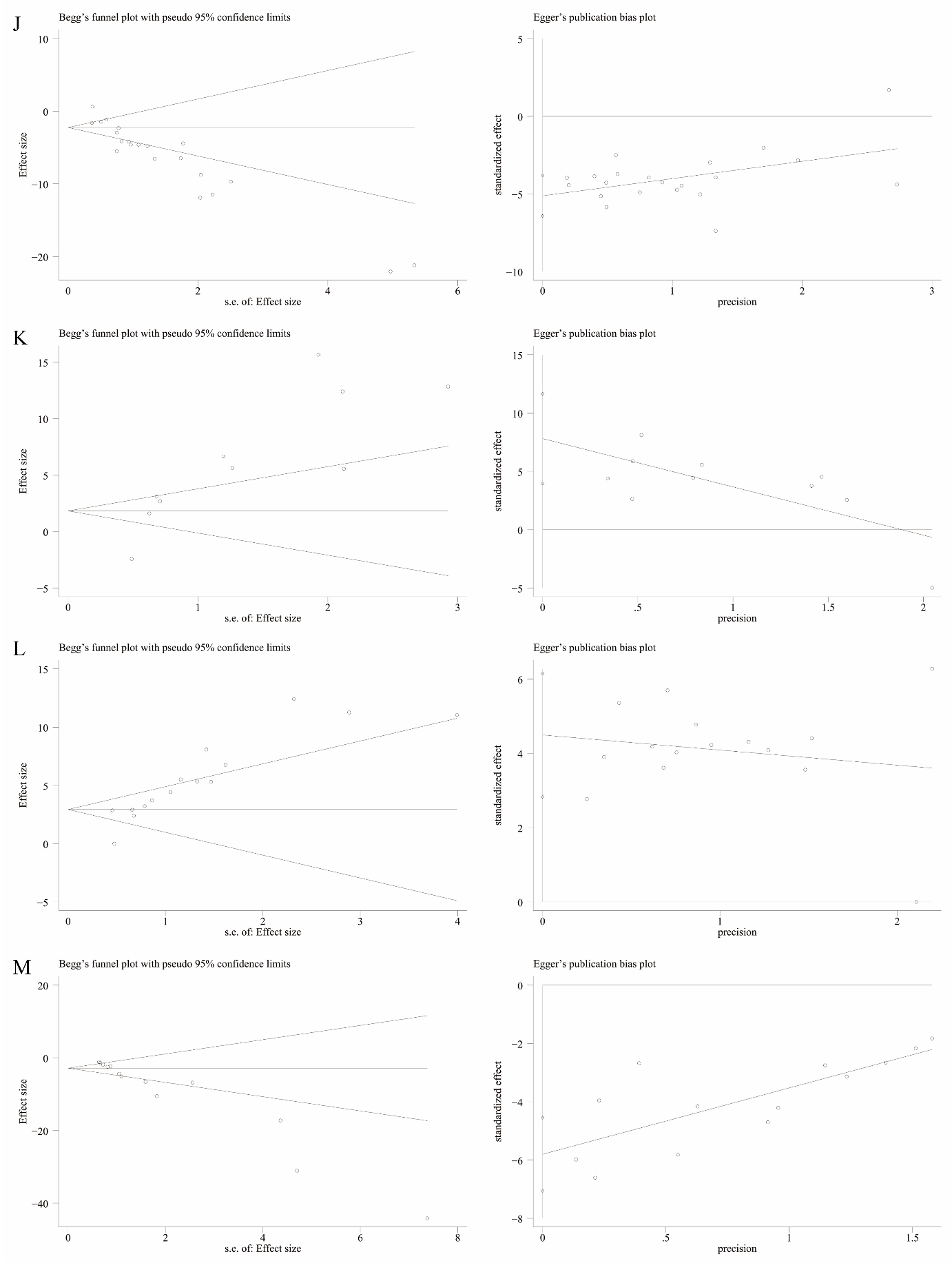
**

Begg’s and Egger’s publication bias plot for (A) degree of liver fibrosis, (B) hydroxyproline, **(C)** TGF-β**, (D)** α-SMA**, (E)** Col1α1, (F) ALT, (G) AST, (H) ALB, (I) ALP, (J) MDA, (K) GSH, (L) SOD and (M) TNF-α.
